# Required *G*_*K*1_ to Suppress Automaticity of iPSC-CMs Depends Strongly on *I*_*K*1_ Model Structure

**DOI:** 10.1016/j.bpj.2019.08.040

**Published:** 2019-09-13

**Authors:** Alan Fabbri, Birgit Goversen, Marc A. Vos, Toon A.B. van Veen, Teun P. de Boer

**Affiliations:** 1 University Medical Center Utrecht, Utrecht, the Netherlands

## Abstract

Human-induced pluripotent stem cells derived cardiomyocytes (hiPSC-CMs) are a virtually endless source of human cardiomyocytes that may become a great tool for safety pharmacology; however, their electrical phenotype is immature: they show spontaneous action potentials (APs) and an unstable and depolarized resting membrane potential (RMP) because of lack of *I*_*K*1_. Such immaturity hampers their application in assessing drug safety. The electronic overexpression of *I*_*K*1_ (e.g., through the dynamic clamp (DC) technique) is an option to overcome this deficit. In this computational study, we aim to estimate how much *I*_*K*1_ is needed to bring hiPSC-CMs to a stable and hyperpolarized RMP and which mathematical description of *I*_*K*1_ is most suitable for DC experiments. We compared five mature *I*_*K*1_ formulations (Bett, Dhamoon, Ishihara, O’Hara-Rudy, and ten Tusscher) with the native one (Paci), evaluating the main properties (outward peak, degree of rectification), and we quantified their effects on AP features (RMP, ${\dot{V}}_{max}$, *APD*_50_, *APD*_90_ (AP duration at 50 and 90% of repolarization), and *APD*_50_/*APD*_90_) after including them in the hiPSC-CM mathematical model by Paci. Then, we automatically identified the critical conductance for *I*_*K*1_ ( *G*_*K*1, *critical*_), the minimally required amount of *I*_*K*1_ suppressing spontaneous activity. Preconditioning the cell model with depolarizing/hyperpolarizing prepulses allowed us to highlight time dependency of the *I*_*K*1_ formulations. Simulations showed that inclusion of mature *I*_*K*1_ formulations resulted in hyperpolarized RMP and higher ${\dot{V}}_{max}$, and observed *G*_*K*1, *critical*_ and the effect on AP duration strongly depended on *I*_*K*1_ formulation. Finally, the Ishihara *I*_*K*1_ led to shorter (−16.3%) and prolonged (+6.5%) *APD*_90_ in response to hyperpolarizing and depolarizing prepulses, respectively, whereas other models showed negligible effects. Fine-tuning of *G*_*K*1_ is an important step in DC experiments. Our computational work proposes a procedure to automatically identify how much *I*_*K*1_ current is required to inject to stop the spontaneous activity and suggests the use of the Ishihara *I*_*K*1_ model to perform DC experiments in hiPSC-CMs.

## Significance

In this work, we aim to contribute a method that will facilitate automated dynamic clamp (DC) experiments in which *I*_*K*1_ is injected in induced pluripotent stem-cell-derived cardiomyocytes (iPSC-CMs). By introducing *G*_*K*1, *critical*_ (minimal *I*_*K*1_ conductance needed to stop automaticity of iPSC-CMs), we are proposing a different approach to setting up DC experiments. These are usually based on the injection of a fixed current density. In contrast, *G*_*K*1, *critical*_ is a parameter that depends on the cell under investigation. Our in silico approach analyzed analogies and differences between *I*_*K*1_ formulations without the confounding factor that can be brought by the variability of iPSC-CMs. It highlighted how much the employed mathematical formulation of *I*_*K*1_ can affect *G*_*K*1, *critical*_ and the action potential waveform in DC experiments.

## Introduction

To be successfully placed on the market, a drug must be effective (i.e., it must be able to hit the desired target at clinically relevant concentrations) and safe (i.e., no side effects that could compromise the function of organs should occur). Insufficient efficacy and safety are responsible for almost 60% of the attrition rate in drug discovery and development (1, 2). In the cardiovascular area, the proarrhythmic potential of a drug is the side effect reporting the highest number of postapproval adverse events (2, 3).

Torsades de pointes is a potentially fatal arrhythmia that may occur when the repolarization of the ventricles is delayed. At the cellular scale, block of the human ether-à-go-go-related gene channels conducting the rapid delayed rectifier potassium current $\left({\text{I}}_{Kr}\right)$ is among the ones responsible for the delayed repolarization of the ventricles, which is detectable in surface electrocardiogram traces as a prolonged QT interval.

The International Committee on Harmonization S7b (preclinical) and the E14 (clinical) guidelines successfully reduced the risk of approving drugs that have the potential to induce torsades de pointes by deprioritizing development of drugs that block human ether-à-go-go related gene channels or cause QT prolongation. However, the guidelines are affected by low specificity (4). To overcome this problem, the comprehensive in vitro proarrhythmic assay (CiPA) initiative suggests an alternative paradigm to assess the safety of a new compound in the preclinical and early clinical stages (4, 5). The paradigm shift consists of the hybrid combination of in vitro and in silico human-specific models (5). The first step in the CiPA paradigm focuses on the evaluation of the functional drug effects on inward currents involved in the plateau phase, such as the long-lasting calcium current (*I*_*CaL*_) and the late sodium current $\left(I_{Na,L}\right)$, and outward currents active during repolarization, such as the slow delayed rectifier potassium current (*I*_*Ks*_), the inward rectifier potassium current (*I*_*K1*_), and the transient outward potassium current (*I*_*to*_). At the second stage, the experimental data are included into an in silico human adult ventricular action potential (AP) model (6) that allows one to investigate the behavior of the affected currents in an integrated environment. Finally, the prediction of the drug’s effects is confirmed or disproved through in vitro experiments using human-induced pluripotent stem cells (hiPSCs) derived cardiomyocytes (hiPSC-CMs). Furthermore, in silico hiPSC-CM models have been successfully employed to assess drug efficacy. Paci et al. (7) showed that mexiletine and ranolazine, two multichannel blockers that target *I*_*Na*_, *I*_*NaL*_, *I*_*CaL*_, and *I*_*Kr*_, are able to recover the AP duration (APD) to physiological values in an LQTS3 hiPSC-CM population. In silico models of hiPSC-CMs also help to quantitatively characterize the differences between hiPSC-CM and adult ventricular cardiomyocytes (7). Indeed, the simulation of the pharmacological block of ion currents showed that the two cells types respond in different ways (8), giving a quantitative description of such a difference.

hiPSC-CMs are a promising tool for drug efficacy and safety testing. Because hiPSCs are obtained by reprogramming adult somatic cells to a pluripotent state, they are a virtually limitless source of cells with the same genome as the donor. Their origin from human adult cells avoids ethical issues associated with the use of human embryonic stem cells, bridge the gap resulting from the use of animal models, and bring personalized medicine closer. However, hiPSC-CMs are characterized by an immature electrophysiological phenotype that limits their employment in drug safety assessment (9). Unlike adult ventricular cardiomyocytes, they show high levels of expression of the HCN gene family (that encodes for the pacemaker current *I*_*f*_) and low levels of the KCNJ2 gene, responsible for *I*_*K*1_. The balance between ion channel types expressed by iPSC-CMs results in a depolarized resting membrane potential (RMP), a slow maximal upstroke velocity $\left({\dot{V}}_{max}\right)$ due to the inactivation of sodium channels, and the absence of the plateau phase. Furthermore, because of the contribution of *I*_*f*_, hiPSC-CMs show spontaneous automaticity (9).

Several studies (10, 11, 12) have demonstrated that compensating for the low levels of *I*_*K*1_ improves the electrophysiological phenotype of hiPSC-CMs, making its phenotype more mature and more relevant for drug safety testing. Apart from attempts to improve differentiation protocols, two direct methods have been used to increase functional expression of *I*_*K*1_ in hiPSC-CMs (e.g., through adenoviral overexpression of *I*_*K*1_ channels (13) or by applying the dynamic clamp (DC) technique to insert virtual *I*_*K*1_ conductance) (14, 15). The DC is a refinement of the traditional patch clamp. It allows one to interface the membrane potential of (one or more) cells with a computer running a real-time simulation of ion channels or gap junctions. In this way, it is possible to create a virtual electrical connection between cells or add a virtual ion channel to the cell membrane through a computational model that describes the time course of that current in response to membrane potential of the connected cell(s). The injected current can be fully described by mathematical equations and parameters. Through the tuning of the parameters, the amount of injected current can be modulated precisely to adapt it to the cell under investigation. The ability to fine-tune ion channel conductance to each cell being tested is essential when adding *I*_*K*1_ to hiPSC-CMs because too little will leave the cells beating spontaneously, whereas too much *I*_*K*1_ can render the cell nonexcitable. Earlier work by Verkerk et al. (11) indeed reported that the injection of *I*_*K*1_ eliminates the spontaneous activity of hiPSC and provides a more physiological phenotype (11). Bett et al. (12) highlighted that hiPSC-CMs respond like adult ventricular CMs to the administration of BayK-8644 only if *I*_*K*1_ is injected. Studies testing DC strategies to mature hiPSC-CMs have almost exclusively used manual patch clamping, but recently, our group successfully implemented DC-based *I*_*K*1_ injection in hiPSC-CM on an automatic patch-clamping platform that is capable of medium- to high-throughput drug screening by recording from up to eight cells in parallel (16, 17).

Fine-tuning of the *I*_*K*1_ conductance (*G*_*K*1_) is an important step in DC experiments, whether manual or automated patch clamping is used. Higher throughput applications however require an automated tuning procedure that can run unattended. In this study, we propose an approach to establish the required *G*_*K*1, *critical*_ in a way that can be implemented on automated patch-clamping platforms. We define $G_{K1,critical}$ as the minimal value of conductance that is sufficient to bring the cell to a stable and hyperpolarized RMP. Identifying *G*_*K*1, *critical*_ is relevant because it allows one to estimate the minimal amount of the injected *I*_*K*1_ for a cell, and it can subsequently be used as a reference value when *G*_*K*1_ is up- or downscaled, facilitating comparison of results obtained from different cells. Next to the value of *G*_*K*1_, the time- and voltage-dependent properties of the used computational model of *I*_*K*1_ affect the AP waveform. Several studies have captured the electrophysiology of *I*_*K*1_ in a computational model, providing descriptions with different levels of detail (6, 12, 18, 19, 20, 21). The choice of the *I*_*K*1_ model used in DC experiments may therefore affect the resulting APs.

In this study, we have employed in silico DC experiments to explore the impact of *I*_*K*1_ on the immature hiPSC-CM electrophysiologic phenotype. Our simulations have the aim to 1) compare the effects on AP waveform of the six different *I*_*K*1_ formulations (6, 12, 18, 19, 20, 21) in the hiPSC-CM computational model developed by Paci et al. (22), 2) establish an algorithm to estimate *G*_*K*1, *critical*_ for the six models, 3) assess how the up- and downscaling of *G*_*K*1_ relative to *G*_*K*1, *critical*_ affects the AP waveform, and 4) study the effects of hyper- or depolarizing prepulses on AP waveform.

## Materials and Methods

### hiPSC-CM model, *I*_*K*1_ formulations and identification of *G*_*K*1, *critical*_

We carried out the in silico experiments using the computational model of hiPSC-CM developed by Paci et al. (22). The Paci model is robustly constrained by experimental data obtained by Ma et al. (20), who electrophysiologically characterized hiPSC-CMs. Furthermore, they quantitatively investigate the mechanisms responsible for the immature electrophysiological phenotype, assessing the effects on spontaneous activity and AP waveform through the replacement of a subset of native currents (*I*_*Na*_, *I*_*to*_, *I*_*Ca,L*_, *I*_*K*1_, *I*_*Kr*_, *I*_*Ks*_) with adult ventricular current formulations from the O’Hara-Rudy model (6). The aforementioned characteristics make it a suitable model for evaluating DC strategies through adding *I*_*K*1_ conductances to hiPSC-CMs.

**Figure 1:**
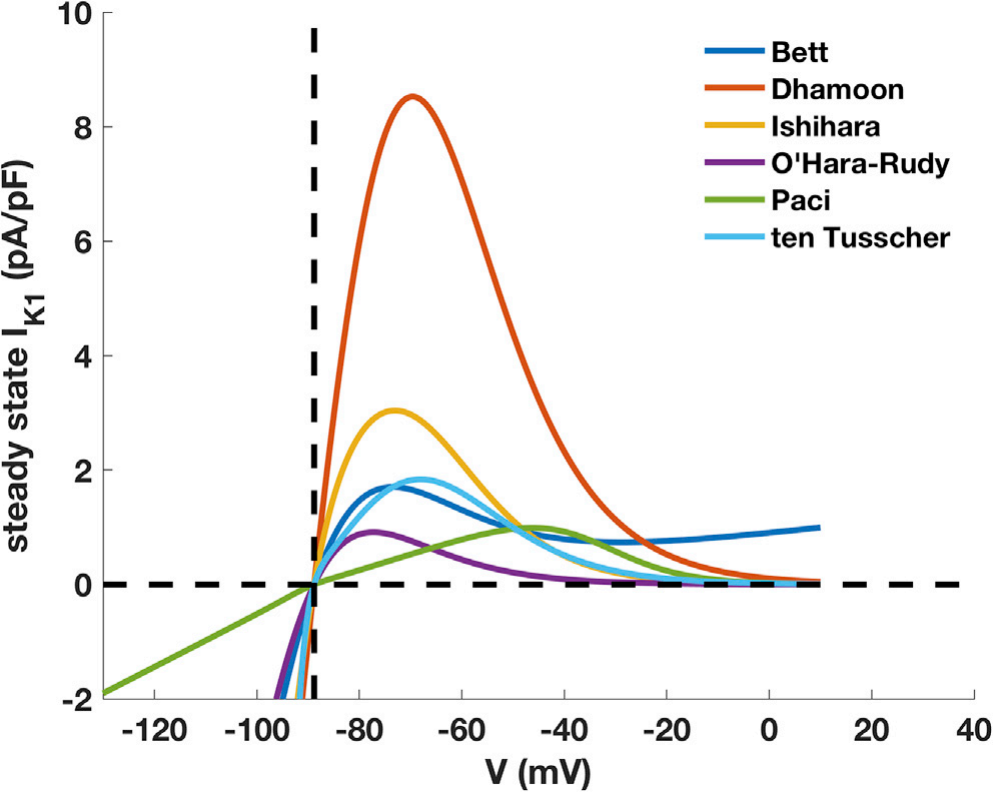
Comparison of the *I*_*K*1_ I-V curves of the six *I*_*K*1_ models for the original (published) *G*_*K*1_ value. *E*_*K*_ is marked by the vertical dashed line. To see this figure in color, go online.

In this study, we have evaluated all *I*_*K*1_ model structures available in the literature, namely, those published by Bett et al. (12), Dhamoon et al. (18), Ishihara et al. (19), Paci et al. (22), O’Hara-Rudy et al. (6), and ten Tusscher et al. (21). The six *I*_*K*1_ formulations mainly differ in the presence (or absence) of time dependency in their kinetics and how the rectification of the outward current is described. A more detailed comparison between the six *I*_*K*1_ formulations is reported in Fig. 1 and Table 1. We set the extracellular potassium ion concentration ${\left[K^{+}\right]}_{o}$ to 5.4 mM and the intracellular concentration ${\left[K^{+}\right]}_{i}$ to 150 mM, setting the reversal potential for the six currents at *E*_*K*_ = −88.8 mV.

**Table 1:** Main Characteristics of the Six *I*_*K*1_ Formulations

*I*_*K*1_ Formulation	Experimental Data Source	Transient	Steady State	Rectification	Conductance	Reference
Bett	adult human ventricular cells	NP	steady state only	voltage dependent	constant	(12, 37)
Dhamoon	guinea pig Kir2.1 in HEK293	NP	steady state only	voltage dependent	constant	(10, 18)
Ishihara	mouse Kir2.1 in HEK 293	v	v	two channel populations sensitive to ${\left[Mg^{2+}\right]}_{i}$ and ${\left[SPM\right]}_{i}$	${\left[K^{+}\right]}_{o}$ dependent	(19, 38)
O’Hara-Rudy	adult human ventricular cells	inactivation gate	v	voltage dependent ${\left[K^{+}\right]}_{o}$ dependent	${\left[K^{+}\right]}_{o}$ dependent	(6)
Paci	hiPS-CM	NP	steady state only	voltage dependent	${\left[K^{+}\right]}_{o}$ dependent G_*K*1_ ×1.1	(20, 22)
ten Tusscher	adult human ventricular and atrial	NP	steady state only	voltage dependent	${\left[K^{+}\right]}_{o}$ dependent	(21, 37)

NP, not present; v, implemented.

To study how the different formulations of *I*_*K*1_ affect the AP waveform, we paced hiPSC-CM cell models at 1 Hz, with a current stimulus of amplitude 15.2 pA/pF and 5 ms of duration, able to elicit AP in all the six models under comparison. The AP waveforms were quantitatively described through five biomarkers: the RMP, maximal speed of depolarization during the upstroke $\left({\dot{V}}_{max}\right)$, the APD at 50 and 90% of repolarization (*APD*_50_ and *APD*_90_), and the ratio *APD*_50_/*APD*_90_, a biomarker to describe triangulation.

The identification of *G*_*K*1, *critical*_ for each *I*_*K*1_ formulation was performed using the bisection algorithm in the unpaced hiPSC-CM. We performed the search in a range from 0 to 10 times the original (published) value. If the membrane potential of the cell was <−40 mV and with a difference between the minimal and maximal values over a period of 50 s that was <1 mV, the cell was defined as quiescent, and the current *G*_*K*1_ was stored as $G_{K1,high}$. On the contrary, if the cell showed spontaneous activity or failed to repolarize, *G*_*K*1_ was stored as $G_{K1,low}$; *G*_*K*1_ employed in the next step was calculated as $\left(G_{K1,high}+G_{K1,low}\right)$/2. The *G*_*K*1_ value that made the cell quiescent and hyperpolarized was then challenged with a single external stimulus and the bisection search was carried out again. The algorithm stopped when the difference between $G_{K1,high}$ and $G_{K1,low}$ was lower than a tolerance set to 0.1% of the original value of *G*_*K*1_.

As a proof of principle, in vitro experiments were carried out to determine *G*_*K*1, *critical*_ in hiPSC-CM in a DC experiment using a custom DC system as described in Goversen et al. (16). Manual patch-clamp recordings were done at 37°C and followed the protocol outlined in Fig. S5. Coverslips with hiPSC-CM were superfused with a bath solution containing NaCl 130 mM, KCl 4 mM, CaCl_2_ 1.8 mM, MgCl_2_ 1.2 mM, NaHCO_3_ 18 mM, HEPES 10 mM, and glucose 10 mM. Pipettes had resistances between 2 and 4 MΩ when filled with a pipette solution containing KCl 10 mM, K-gluconate 125 mM, CaCl_2_ 0.6 mM, MgCl_2_ 2 mM, HEPES 5 mM, sucrose 30 mM, Na_2_ ATP 4 mM, and EGTA 5 mM. Liquid junction potential (+13.8 mV) was calculated using pCLAMP 10 and corrected a priori.

The effect of increasing or decreasing *G*_*K*1_ was investigated by running AP simulations paced at 1 Hz, using values for *G*_*K*1_ in a range from 0- to 10-fold *G*_*K*1, *critical*_, increasing *G*_*K*1_ of 0.25 × $G_{K1,critical}$.

The last protocol aimed to reproduce the experiment by Ishihara et al. (23), using hyper-/depolarizing current prepulses. Before the pacing stimulus that triggered the AP, 50 ms current prepulses of 5 and −0.5 pA/pF were applied to respectively hyperpolarize or depolarize the voltage membrane. The combination of the prepulses and the pacing stimulus was administered at 1 Hz for 30 s.

### Comparing the influence of cell-to-cell variation between iPSC-CMs and between *I*_*K*1_ formulations

The Paci 2013 model is based on the data collected by Ma et al. (20) using hiPSC-CMs. To address the variability between cells that is encountered in experiments, we identified *G*_*K*1, *critical*_ for each *I*_*K*1_ formulation under investigation in a population of 22 cell-specific iPSC-CM models, published earlier by Lei et al. (24). In brief, Lei et al. (24) adapted the Paci 2013 model on the base of voltage clamp experiments carried out on hiPSC-CMs, by scaling the maximal conductance (S/F) of *I*_*Na*_ (×0.69), *I*_*CaL*_ (×0.80), *I*_*Ks*_, and the maximal activity of *I*_*NaCa*_ (pA/pF) (tailored for each cell, the values are reported in Table 2). Next, we assessed the variability of the AP morphology while pacing the cell-specific models at 1 Hz.

**Table 2:** AP Waveform Parameters of hiPSC-CM Models Including the six *I*_*K*1_ Formulations at *G*_*K*1, *original*_

Model	RMP (mV)	${\dot{V}}_{max}$ (V/s)	*APD*_50_ (ms)	*APD*_*9*0_ (ms)	*APD*_50_/*APD*_90_ (−)
Paci (unpaced)	−76.9	24.9	366	487	0.75
Bett	−86.8	157.5	58	153	0.38
Dhamoon	−88.1	160.9	101	133	0.76
Ishihara	−87.8	157.9	84	163	0.57
O’Hara-Rudy	−20.4	NA	NA	NA	NA
Paci	−77.2	57.9	302	453	0.67
ten Tusscher	−86.1	136.4	258	431	0.60

The *APD*_50_/*APD*_90_ ratio provides a quantification of the shape of the AP waveform.

### Hardware and software

The Paci-based hiPSC-CM models including the six *I*_*K*1_ formulations were encoded in CellML and solved using OpenCOR (version 0.6) (25) and on the Cardiac Electrophysiology Web Lab (available at https://travis.cs.ox.ac.uk/FunctionalCuration/) (26). Simulations done using OpenCOR were performed on a macOS High Sierra (10.13.6) Apple computer (Apple, Cupertino, CA) equipped with 2.9 GHz quad-core Intel Core i7 (Intel, Santa Clara, CA). We used a variable step method for stiff problems (backward differentiation formula) implemented in the CVODE library. The currents and the state variables of the models were stored and displayed with a 0.1 ms time step. The cell models achieved steady-state conditions when the difference of the norm of the state variable vector at the beginning and at the end of the AP was lower than 10^−6^. The extraction of AP waveform features and plots were performed in MATLAB (release R2018a; The Mathworks, Natick, MA).

## Results

### hiPSC-CM AP waveform is strongly influenced by *I*_*K*1_ model structure

A first comparison between the six *I*_*K*1_ formulations was made by plotting the steady-state current generated by the models at voltages between −120 and +10 mV (see Fig. 1). The Dhamoon model shows the highest outward peak (8.5 pA/pF at −69.5 mV), whereas *I*_*K*1_ from the O’Hara-Rudy model shows the lowest outward peak current density (0.91 pA/pF at −77.1 mV). The Paci model, constructed using experimental data obtained from immature hiPSC-CMs, is remarkably different from the other models, which are based on experimental data from adult myocytes or heterologous expression systems, and reaches the outward peak (0.99 pA/pF) at more depolarized potentials (−46.1 mV). The model structures also differ in the rectification: the Dhamoon model shows *I*_*K*1_ density close to zero at depolarized potentials ($V_{m}$ > 0 mV), whereas the O’Hara-Rudy model already reaches small current densities around −40 mV. The Bett model differs markedly at positive potentials, showing a clear positive linear growth.

Next, we simulated paced APs using the Paci models incorporating the six *I*_*K*1_ models. Analysis of AP waveform allows quantification of the effects of the *I*_*K*1_ formulations. The cell models that include the Dhamoon and the Paci *I*_*K*1_ formulations show the most hyperpolarized and depolarized RMPs and the fastest and the lowest ${\dot{V}}_{max}$ (−88.1 mV, 160.9 V/s, and −77.2 mV, 57.9 V/s, respectively). The key role played by *I*_*K*1_ in repolarization becomes clear when comparing *APD*_50_ and *APD*_90_ values. On one hand, the activity of *I*_*K*1_ during the plateau and phase 3 of the Bett model leads to shorter AP durations (*APD*_50_ = 58 ms, *APD*_90_ = 153 ms). On the other hand, in the model including the O’Hara-Rudy formulation, the low amplitude of *I*_*K*1_ is not sufficient to successfully repolarize the membrane potential that settles to a stable but depolarized RMP (−20.4 mV), as also reported by Paci et al. (22).

Calculating the ratio of *APD*_50_ and *APD*_90_ ( *APD*_50_/*APD*_90_) gives some additional insight about the repolarization course. Values close to 0.5 result from APs with a triangular shape for which the plateau phase is virtually absent and the repolarization is monotonic, whereas values close to 1 occur when phase 3 is steep, and thus the final phase of repolarization is fast. The Bett model shows a value (*APD*_50_/*APD*_90_ = 0.38) that is coherent with a triangular waveform, whereas the Dhamoon model has *APD*_50_/*APD*_90_ = 0.76, consistent with a steep phase 3 repolarization. The Ishihara, the Paci, and ten Tusscher models show *APD*_50_/*APD*_90_ > 0.5 (0.57, 0.67, 0.60, respectively).

**Figure 2:**
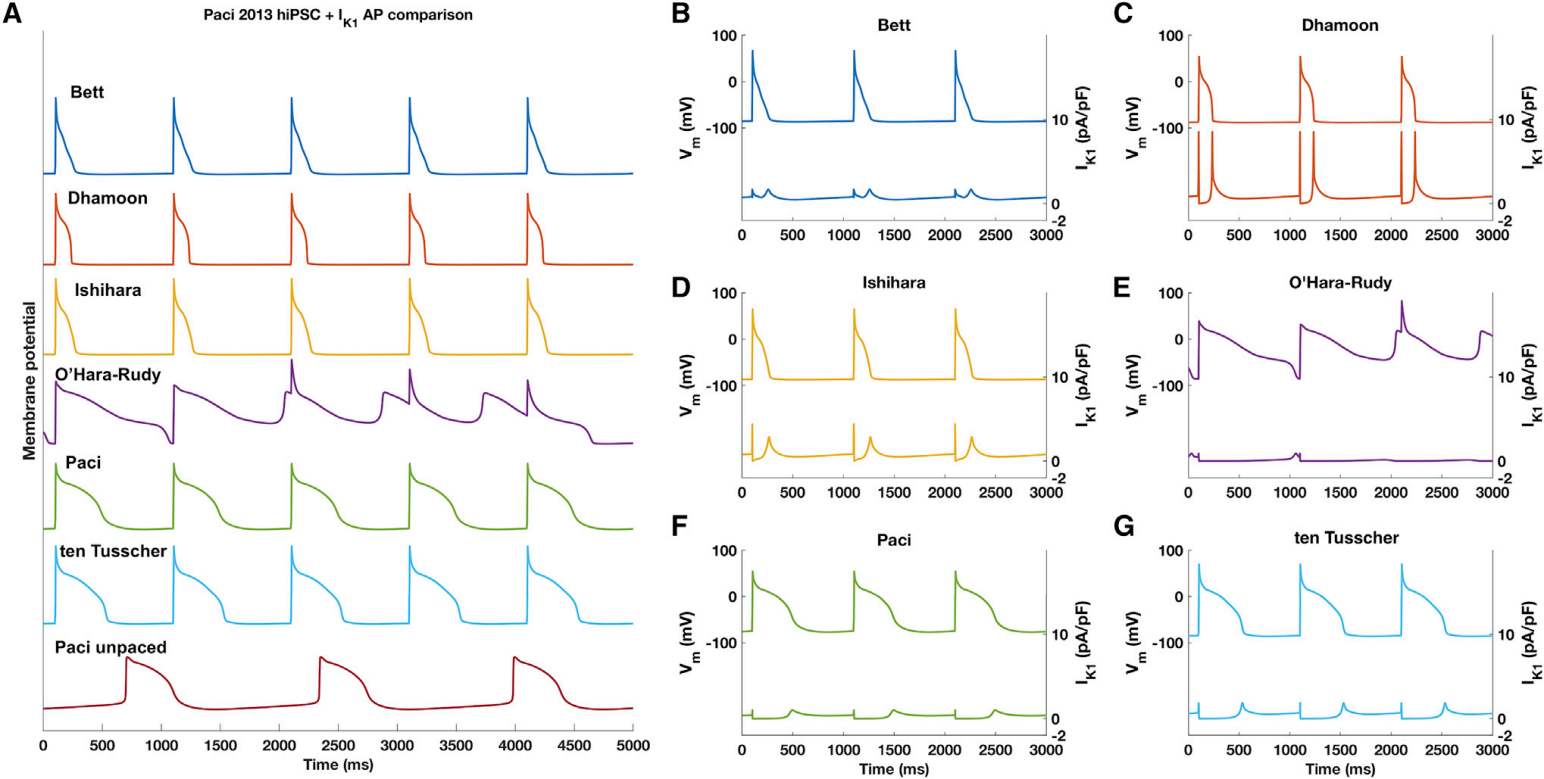
(*A*) AP waveforms elicited by pacing the Paci hiPSC-CM model incorporating the six different *I*_*K*1_ formulations at 1 Hz, with the original *G*_*K*1_. The bottom trace displays the original hiPSC-CM Paci model without pacing, which shows the spontaneous activity that is typical of hiPSC-CMs. (*B*–*G*) *V*_*m*_ and *I*_*K*1_ time courses of the hiPSC-CM cell models including the six *I*_*K*1_ formulations are shown. The model including O’Hara-Rudy *I*_*K*1_ formulation failed to repolarize after 70 s; here, we report the transition from successful to failing repolarization (fourth trace in *A* and *E*). To see this figure in color, go online.

To get a better understanding of the influence of the six models on emerging AP waveforms, we compared the AP waveforms and underlying *I*_*K*1_ current profiles (see Fig. 2). This showed a clear effect of *I*_*K*1_ model structure on the AP waveform. The Dhamoon *I*_*K*1_ shows the largest outward peak (8.5 pA/pF), as expected from the steady-state I-V characteristic. The Bett current is active throughout all AP phases but mostly during the plateau phase (∼1 pA/pF), during which the other models produce currents of negligible amplitude. The Ishihara *I*_*K*1_ contributes to the early phase 3 of the AP, starting earlier during the repolarization phase than the Dhamoon, Paci, and ten Tusscher *I*_*K*1_ models. The Dhamoon model shows the largest peak (8.5 pA/pF) during late repolarization at which it contributes to a fast final repolarization toward the RMP. The Dhamoon and the Ishihara models both bring the RMP (−88.1 and −87.8 mV, respectively) close to *E*_*K*_ (−88.8 mV) by conducting a current density of ∼0.8 pA/pF. The lowest *I*_*K*1_ density during phase 4 is generated by the Paci model, which also shows the least hyperpolarized RMP (−77.2 mV).

### Scaling relative to *G*_*K*1, *critical*_ facilitates comparison of *I*_*K*1_ models or cells

The critical value of *G*_*K*1_ (*G*_*K*1, *critical*_ ) is an estimation of the minimal number of *I*_*K*1_ channels that is required on the cell membrane to suppress automaticity, to make the cell 1) quiescent, 2) with an RMP that is stable and close to *E*_*K*_, even when the cell is perturbed with an external stimulus. After establishing *G*_*K*1, *critical*_ for all six *I*_*K*1_ models, we observed two different behaviors. When incorporating the Bett, Dhamoon, Ishihara, or ten Tusscher models, the hiPSC-CM models become quiescent with *G*_*K*1_ values that are lower than those in the original *I*_*K*1_ models (see Table 3), with a decrease of 31.4, 87, 60.4, and 41.3%, respectively. On the other hand, the Paci and O’Hara-Rudy models needed an increase of $G_{K1}$ (+53.8 and +46.6%, respectively) to stabilize RMP; this means that these two models do not provide enough *I*_*K*1_ in their original formulation to stop the automaticity. Time course of membrane potential and IK1 are depicted in Fig. S2.

**Table 3:** AP Features of the 1 Hz Paced hiPSC-CM Models for *G*_*K*1_ = *G*_*K*1, *critical*_

Model	*G*_*K*1, *original*_ (S/F)	*G*_*K*1, *critical*_ (S/F)	*ΔG*_*K*1_ (%)	RMP (mV)	${\dot{V}}_{max}$ (V/s)	*APD*_50_ (ms)	*APD*_90_ (ms)	*APD*_50_/*APD*_90_ (−)
Bett	1000	685.8	−31.4	−85.9	145.0	85	242	0.35
Dhamoon	1000	129.4	−87	−84.0	112.8	301	541	0.56
Ishihara	2500	989.7	−60.4	−85.9	132.3	238	458	0.52
O’Hara-Rudy	190.8	279.8	+46.6	−86.7	112.8	349	686	0.51
Paci	28.149	43.3	+53.8	−80.1	85.1	242	367	0.66
ten Tusscher	5405	3170	−41.3	−83.7	108.2	306	546	0.56

*ΔG*_*K*1_, variation (in %) between the original and critical value of *G*_*K*1_; *G*_*K*1, *critical*_, critical value of Kir2.1 conductance that brings RMP to stable and hyperpolarized values; *G*_*K*1, *original*_, default value of Kir2.1 conductance.

**Figure 3:**
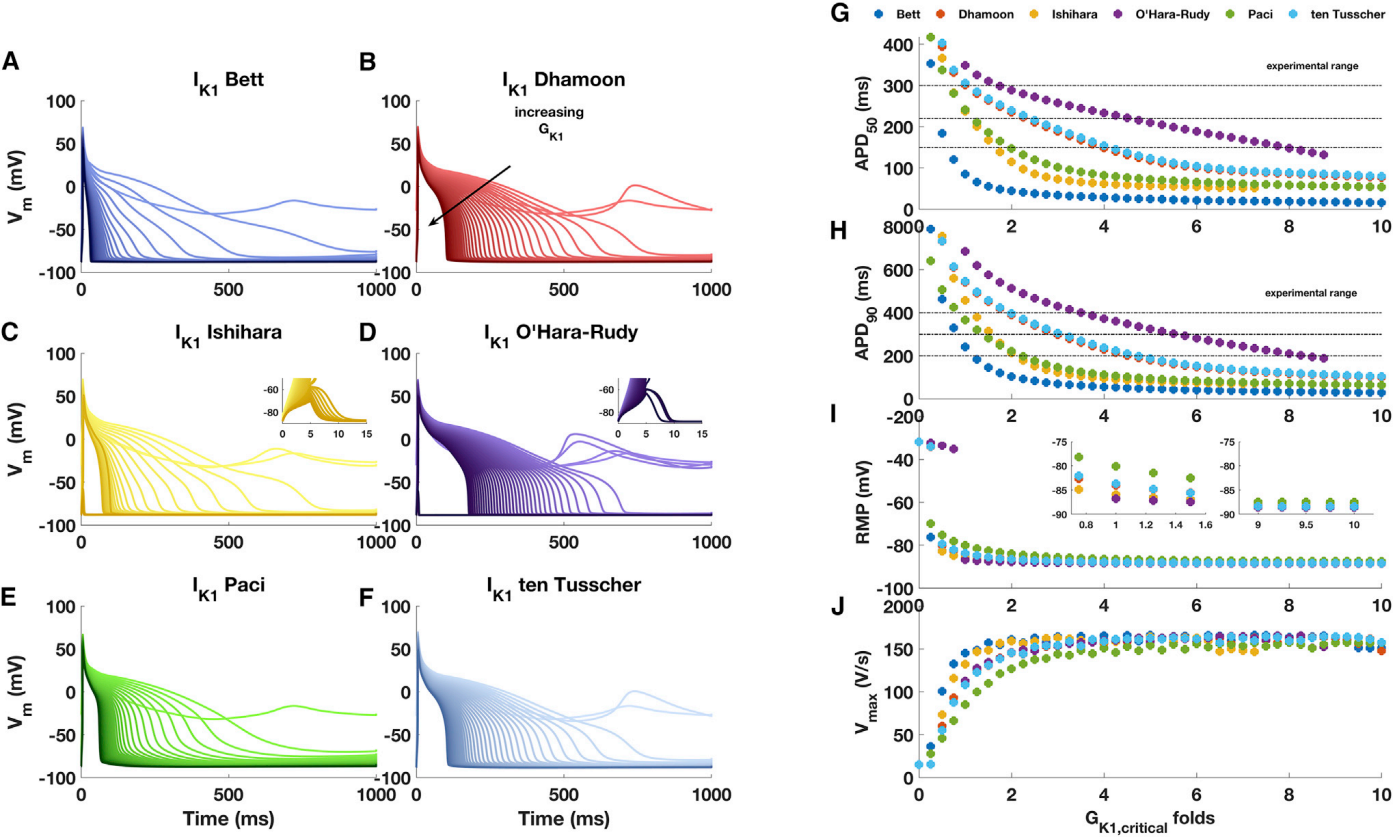
(*A*–*F*) AP waveforms at varying *G*_*K*1_ formulations. Note how a *G*_*K*1_ value that is too large prevents the pacing stimulus from triggering an AP for cell models that include Ishihara et al. (19) and O’Hara-Rudy et al. (6) *I*_*K*1_ (insets in *C* and *D*). Light colors code for low *G*_*K*1_ values, and dark colors code for high *G*_*K*1_ values. (*G*–*J*) AP features (*APD*_50_, *APD*_90_, RMP, and ${\dot{V}}_{max}$) extracted from the membrane potential time courses are shown. Simulations were run using *G*_*K*1_ values ranging from 0 (no *I*_*K*1_) to 10×$G_{K1,critical}$ and increase with 0.25×$G_{K1,critical}$ step. The experimental range for *APD*_50_ and *APD*_90_ refers to adult healthy cardiomyocytes from Britton et al. (29). To see this figure in color, go online.

Fig. 3, *A–F* depicts the AP waveform with *G*_*K*1_ values ranging between 0 and 10× *G*_*K*1, *critical*_. The downscaling of *G*_*K*1_ resulted in longer APDs and a depolarized RMP when the cells were able to repolarize. Progressively increasing *G*_*K*1_ resulted in a faster repolarization process with a smaller *APD*_50_ and *APD*_90_ (Fig. 3, *G* and *H*). A higher amount of *I*_*K*1_ is also responsible for a hyperpolarized and stable RMP (Fig. 3 *I*) that gets close to *E*_*K*_. The inset in Fig. 3 *I* shows that for a conductance corresponding to *G*_*K*1, *critical*_, RMPs are still quite different: the hiPSC-CM model including the Paci formulation is the most depolarized (−80.1 mV), whereas the model including the O’Hara-Rudy formulation is the most hyperpolarized (−86.7 mV).

For all *I*_*K*1_ models, the upstroke velocity ${\dot{V}}_{max}$ (Fig. 3 *J*) increased with increasing *G*_*K*1_ values from ∼0.5 to 2× *G*_*K*1, *critical*_ and then stabilizes at values around 150 V/s for larger *G*_*K*1_ values. For *G*_*K*1_ > 7.5× *G*_*K*1, *critical*_ and > 9.25× *G*_*K*1, *critical*_, respectively, the pacing stimulus was no longer sufficient to trigger an AP when using the Ishihara or the O’Hara-Rudy models (Fig. 3, *C* and *D*, see *insets*). Together with *G*_*K*1, *critical*_, this behavior suggests that there is a range for the amount of *I*_*K*1_ that should be injected into the cell, providing indications for in vitro experiments.

### G_K1, critical_ shows limited variability between cell-specific iPSC-CM models

**Figure 4:**
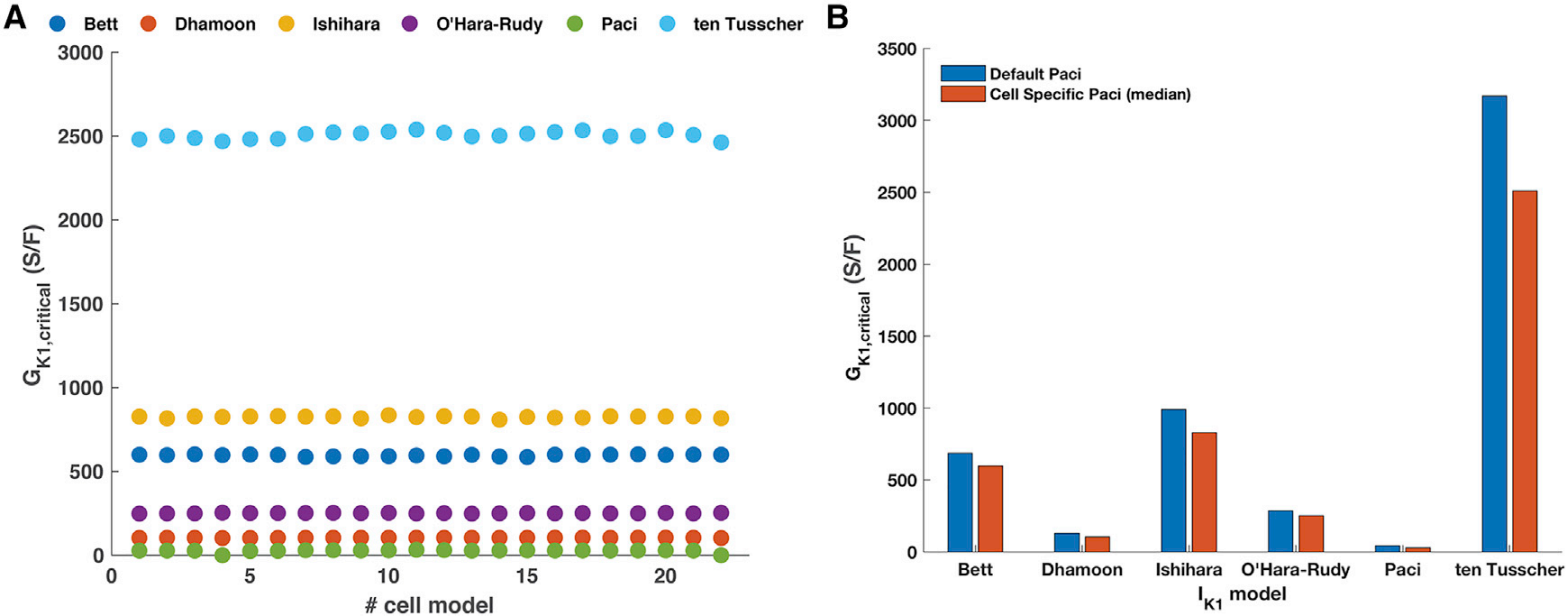
(*A*) *G*_*K1, critical*_ values of each of the 22 cell specific Paci models, including the *I*_*K1*_ formulations according to Bett et al. (12), Dhamoon et al. (18), Ishihara et al. (19), Paci et al. (22), O’Hara-Rudy et al. (6), and ten Tusscher et al. (21). (*B*) Comparison between *G*_*K1, critical*_ identified in the default Paci model (*blue bars, left*) and the median *G*_*K1, critical*_ for the cell specific Paci models (*red bars, right*). Notably, *G*_*K1, critical*_ varies more between *I*_*K1*_ formulations than between cells. To see this figure in color, go online.

Variation in AP waveform between hiPSC-CM is well known, and this may influence the *G*_*K*1, *critical*_ value required in a DC experiment for maintaining quiescence of a particular cell. To test this, we employed 22 cell-specific iPSC-CM models, tailored on the Paci 2013 model, with variations in ion currents based on voltage clamp data (24). For each cell-specific model, we identified *G*_*K*1, *critical*_ using the six *I*_*K*1_ formulations we wanted to assess. Fig. 4 illustrates the range of the 22 *G*_*K*1, *critical*_ values obtained for each *I*_*K*1_ formulation. *G*_*K*1, *critical*_ did not follow a normal distribution; therefore, we described them through the median and 25th and 75th percentiles. Median *G*_*K*1, *critical*_ was 598.6 ($I_{K1}$ according to Bett), 104.2 (Dhamoon), 827.2 (Ishihara), 28.2 (Paci), 251.2 (O’Hara-Rudy), and 2510 S/F (ten Tusscher). Within the six populations of cell-specific models, we found a limited range for *G*_*K*1, *critical*_ : the population including the *I*_*K*1_ according to Paci reported the largest variation of the 25th and 75th percentile from the median *G*_*K*1, *critical*_ (−2.47 and +2.82%, respectively), whereas the models including the other *I*_*K*1_ formulations showed variations lower than 1%. Table S1 reports in detail the cell-specific *G*_*K*1, *critical*_ identified for each cell.

To test if our findings with the cell-specific models were reflected in in vitro experiments, a small set of DC experiments was done using hiPSC-CM. We injected *I*_*K*1_ using the formulation by Ishihara and determined *G*_*K*1, *critical*_ (see Figs. S5 and S6). In in silico experiments, conditions are ideal, and we were able to check if the membrane potential was stable and in steady state during a 50-s-long time window for many tested *G*_*K*1_ values. During in vitro experiments, membrane potential is less stable because of noise, and testing many 50-s iterations of the bisection algorithm takes more time than typically feasible in patch-clamp experiments. For the aforementioned reasons, we did not implement a bisection algorithm but instead started from a large *G*_*K*1_ value (5000 S/F), which was decreased in steps of 500 S/F. The average *G*_*K*1, *critical*_ was 2750 ± 660 S/F (*n* = 4), with values ranging from 1000 to 4000 S/F, confirming the cell-specific nature of the *G*_*K*1, *critical*_ parameter. In three additional cells, slightly more than 5000 S/F was needed to suppress minimally remaining automaticity, but the correct value could not be obtained before the experiment expired.

The comparison between the median *G*_*K*1, *critical*_ found in the cell-specific models and the one identified in the initial models used earlier (Fig. 4 *B*) helps to get more insight in the differences between cells. In the cell-specific models, for all the included *I*_*K*1_ formulations, the *G*_*K*1, *critical*_ value observed was lower than the one identified in the initial Paci 2013 models. The distance between the initial Paci 2013 and the cell-specific models was the highest in the models including *I*_*K*1_ according to Paci (−38.2%), whereas it reached −12.3% when the models included the O’Hara-Rudy *I*_*K*1_ formulation. The difference among *G*_*K*1, *critical*_ in the other *I*_*K*1_ formulations was within the boundaries set by Paci and O’Hara-Rudy *I*_*K*1_ models.

**Figure 5:**
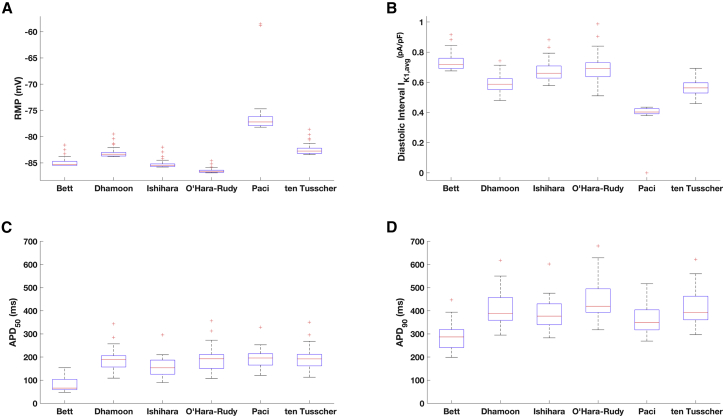
Main AP features of the cell-specific models paced at 1 Hz. (*A*) RMPs are within in a limited range. The population of models including *I*_*K*1_ according to Paci et al. (22) showed a less negative RMP (RMP_median_ = −77.4 mV). (*B*) The average *I*_*K*1_ density during the last 50 ms of the diastolic interval (before the stimulus) is shown; because of the less negative RMP, the current density is lower in the population that includes the Paci *I*_*K*1_. (*C* and *D*) *APD*_50_ and *APD*_90_ show a higher variability within the populations. To see this figure in color, go online.

We further assessed the behavior of the cell-specific models by eliciting APs with external stimuli at 1 Hz, using the cell-specific *G*_*K*1, *critical*_. RMP was hyperpolarized, close to *E*_*K*_ and within a limited range (Fig. 5 *A*; Fig. S3), consistent with the low variability of *G*_*K*1, *critical*_. *I*_*K*1_ contributes to stabilization of RMP. We compared the contribution of the *I*_*K*1_ formulations by measuring the average *I*_*K*1_ current density during the diastolic interval, just before the external stimulus (Fig. 5 *B*). The amount of current is comparable between the six *I*_*K*1_ formulations but consistent with the less negative RMP, the population that includes the Paci *I*_*K*1_ model reported a lower average *I*_*K*1_ density during the diastolic interval. *APD*_50_ and *APD*_90_ showed stronger variability (Fig. 5, *C* and *D*), which is consistent with the cell-specific variations in *I*_*Ks*_ and *I*_*NaCa*_ current densities. The population including *I*_*K*1_ according to Bett showed the shortest *APD*_90_ (287 ms), whereas the others are included within 300 and 450 ms. For a detailed overview of the AP waveform of the cell-specific models, see Fig. S3 and Table S1.

### Reproducing the effect of prepulses on APD requires time dependence of *I*_*K*1_

**Figure 6:**
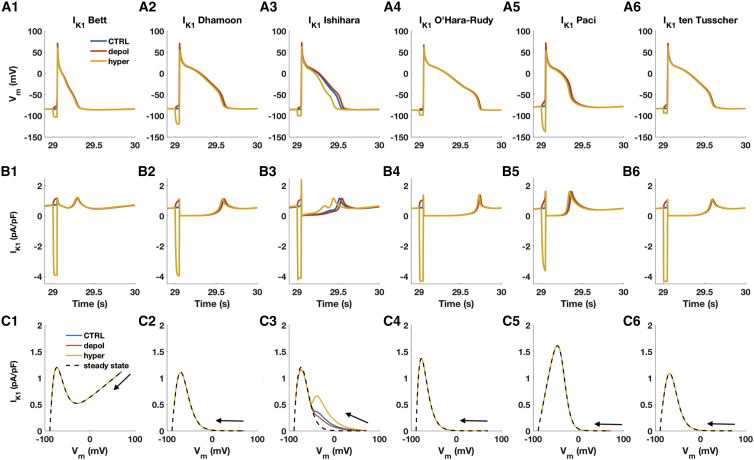
(*A*1–6 and *B*1–6) Membrane potential and *I*_*K*1_ waveforms for the hiPSC-CM models including the six *I*_*K*1_ formulations during the last beat. The cell models were paced at 1 Hz for 30 s. The hyperpolarizing and the depolarizing current prepulse had an amplitude of 5 and −0.5 pA/pF, respectively, and were applied for 50 ms before the AP triggering pulse. Note that only the model including the Ishihara *I*_*K*1_ formulation shows significant effects on AP duration (APD). (*C*1–6) *I*_*K*1_ phase plots of *I*_*K*1_ plotted against membrane potential during repolarization (*blue*, *red*, and *yellow solid lines*) and in steady state (*black dashed lines*) are shown. Arrows point from the start of repolarization toward the end. The Ishihara et al. (19) *I*_*K*1_ formulation provides an outward current at more depolarized potential during the repolarization phase. Such outward current contribution is not present in steady-state conditions in which *I*_*K*1_ density is much smaller at depolarized potentials. No differences between dynamic IV curves during repolarization and during voltage clamp protocol (steady-state conditions) were observed in the other models. To see this figure in color, go online.

Although most *I*_*K*1_ models do not include a time constant in the equations describing rectification, experimental data have demonstrated an effect of time dependence of *I*_*K*1_ channel rectification (23), as brief prepulses given before the pacing stimulus have been shown to affect APD in guinea pig ventricular cardiomyocytes. Specifically, a hyperpolarizing prepulse caused faster repolarization, that is, a shorter APD, whereas a depolarizing prepulse led to APD prolongation. It was demonstrated that *I*_*K*1_ was the current underlying this behavior because of the different availability of open channels at hyperpolarized/depolarized membrane potentials. We tested whether prepulses affect the APD in the initial hiPSC-CM models with the six different *I*_*K*1_ models because contribution of *I*_*K*1_ to variation in repolarization duration can affect the outcome of DC experiments aimed testing proarrhythmic properties of drugs. Fig. 6, *A*1–6 and *B*1–6 depict the AP waveforms and *I*_*K*1_ profiles of the last beat of a train of paces of the six hiPSC-CM models. At first sight, it is clear that the hiPSC-CM model including the Ishihara *I*_*K*1_ formulation is the only one to show significant effects on AP, as the hyperpolarizing current prepulse caused *APD*_90_ prolongation (+6.5%), whereas the depolarizing prepulse shortened *APD*_90_ (−16.3%) with respect to the AP elicited with no prestep, reproducing qualitatively the experimental data. The effects observed in the other AP models were negligible (see Table S2). For the O’Hara-Rudy model, this was surprising because this *I*_*K*1_ model does include a time constant in the description of rectification by including an instantaneous rectification factor $\left(R_{K1,\infty }\right)$ and an inactivation gating variable $\left(x_{K1,\infty }\right)$. In the simulations of hiPSC-CM with the O’Hara-Rudy *I*_*K*1_, closer inspection of the behavior of these parameters showed that *x*_*K*1_ was only minimally affected by the prepulses, ranging between 1 and 0.993 (minimal value obtained with the hyperpolarizing prepulse; see Fig. S4, *G–I*). Fig. 6 *C*1–6 shows *I*_*K*1_ phase plots in which *I*_*K*1_ density is plotted against *V*_*m*_ during repolarization, similar to a phase diagram depicting $\dot{V_{m}}$ versus *V*_*m*_. The Ishihara formulation provided different amounts of *I*_*K*1_ in response to the prepulses. In dynamic conditions, the *I*_*K*1_ phase plots are different from *I*_*K*1_ in steady state, showing a larger amount of *I*_*K*1_ available also at more depolarized potentials. In particular, when the cell is preconditioned with a hyperpolarizing prepulse, it is possible to notice a further smaller *I*_*K*1_ peak (0.66 pA/pF) at −38.5 mV responsible for the APD shortening. In contrast, no appreciable differences were observed with the other models in which the *I*_*K*1_ phase plots overlapped the steady-state IV curve. Therefore, the *I*_*K*1_ formulation that we consider the most suitable to implement in DC experiments is the one according to Ishihara et al. (19).

## Discussion

hiPSC-CMs are a promising tool for drug safety screening and to study cardiac arrhythmia mechanisms. They represent a pillar in the CiPA initiative and allow researchers to study the effects of drugs on AP morphology in an integrated cellular environment with similarities to human adult cardiomyocytes. The main characteristic that hampers their application in assessing drug safety is their spontaneous activity that leads to an unstable and depolarized RMP (*V*_*m*_ > −60 mV). Previous studies demonstrated that is possible to improve the “immature” phenotype of hiPSC-CM through the overexpression of *I*_*K*1_ either by virally overexpressing *I*_*K*1_ channels in the cells (13) or through “electronic expression” using the DC technique (11, 12, 16, 27). DC is a powerful technique because it is able to mimic *I*_*K*1_ carried by biological ion channels. Although it is not able to reproduce the molecular interactions that real *I*_*K*1_ channels can participate in, DC does allow fine control of the amount of *I*_*K*1_ added and allows the experimentalists to change it “on the fly,” adapting it to characteristics (e.g., maturity, AP waveform, cell size) of the cell under investigation.

Our study aims to answer two main questions. 1) How much *I*_*K*1_ has to be injected to stop the automaticity with a stable RMP? 2) Which is the most suitable *I*_*K*1_ mathematical formulation to employ during in vitro DC experiments? We addressed these two questions through a computational approach, using the Paci et al. model (22) as a virtual hiPSC-CM and simulating DC experiments. To this end, we 1) compared a subset of *I*_*K*1_ formulations present in literature and assessed how they affect the AP waveform of the simulated hiPSC-CM, 2) identified the minimal amount of virtual *I*_*K*1_ channels needed to stop the automaticity through the parameter $G_{K1,critical}$, 3) investigated the effects on AP waveform features of down-/upscaling of *I*_*K*1_, and 4) tested the capability of the *I*_*K*1_ formulations to reproduce experimental data reporting APD dependency on hyper- or depolarizing prepulses through an *I*_*K*1_-specific mechanism.

### *I*_*K*1_ formulation and its effects on AP waveform

The comparison of the steady-state I-V curves highlights the differences between the *I*_*K*1_ formulations under investigation. The original Paci et al. (22) model includes *I*_*K*1_, characterized by a low outward peak current at depolarized potentials (∼−46 mV). The other *I*_*K*1_ models derived from adult cardiomyocyte models show substantial differences in the outward peak current or in the degree of rectification. The peak outward current density ranges between 1 (O’Hara-Rudy et al. (6)) and ∼8 pA/pF (Dhamoon et al. (18)). The *I*_*K*1_ formulation according to Bett et al. (12) shows a low degree of rectification, with substantial *I*_*K*1_ density also at more depolarized potentials. The Ishihara et al. (19) *I*_*K*1_ formulation has a more complex mathematical formulation compared to the other models and quantitatively describes how the block exerted by intracellular cations as Mg^2+^ and polyamines can affect the ion channel gating.

In silico experiments allowed us to assess the effects of the aforementioned properties on the AP waveform, avoiding disturbances caused by the high variability of AP waveform in real hiPSC-CMs (11). When the evaluated *I*_*K*1_ formulations replace the native one in the Paci et al. (22) model, RMP becomes stable and hyperpolarized. This holds true for models with all tested *I*_*K*1_ formulations when paced at 1 Hz, but the *I*_*K*1_ formulated according to O’Hara-Rudy et al. (6) leads to stable but depolarized RMP (−20.4 mV), as showed by Paci et al. (22). These results highlight that the amount of *I*_*K*1_ provided by the O’Hara-Rudy et al. (6) model in the original formulation is not enough to stabilize RMP to hyperpolarized values. Simulations confirmed the link between hyperpolarized RMP and ${\dot{V}}_{max}$; the overexpression of *I*_*K*1_ brings the cell to a different working point (i.e., the gating variables are in a different state), unveiling *I*_*Na*_ that is responsible for a faster upstroke. A strong *I*_*K*1_ is responsible for a faster repolarization (see the steep phase 3 in the hiPSC-CM model including the formulation according to Dhamoon et al. (18)), and this characteristic, together with a strong rectification, leads to *APD*_50_/*APD*_90_ ratios >0.5. On the other hand, the low degree of rectification in the Bett et al. (12) formulation is responsible for a substantial current (∼1 pA/pF) at depolarized potentials that abolished the plateau phase. Our results are in agreement with the study by Verkerk et al. (11) that included in the Paci et al. (22) model the *I*_*K*1_ formulations according to Meijer van Putten et al. (27), Bett et al. (12), and Rocchetti et al. (28). In their simulations, they reported a stable and hyperpolarized RMP, a faster ${\dot{V}}_{max}$, and a faster repolarization. As reported in our simulation, they also showed that Bett et al. (12) *I*_*K*1_ dramatically shortened the *APD*.

### How much *I*_*K*1_ should be injected to suppress spontaneous activity?

Earlier studies employing DC to inject *I*_*K*1_ in hiPSC-CMs have typically standardized the amount of added *I*_*K*1_ relative to cell capacitance (e.g., a fixed peak outward current density). In this study, we have described an alternative approach based on the critical *G*_*K*1_, defined as the parameter that depicts the minimal number of *I*_*K*1_ channels on the cell membrane needed to bring the membrane potential to hyperpolarized and stable values, thereby suppressing the spontaneous activity. Our DC experiments in hiPSC-CM indeed confirm that different cells have a different *G*_*K*1, *critical*_ value (Fig. S6).

In our simulations, the identification of *G*_*K*1, *critical*_ for each model showed a substantial decrease (up to −87%) for the *I*_*K*1_ formulations according to Bett et al. (12), Dhamoon et al. (18), Ishihara et al. (19), and ten Tusscher et al. (21). AP waveform analysis of the simulated AP highlights the correlation between *G*_*K*1_ and RMP: higher *G*_*K*1_ leads to more hyperpolarized RMP (see also the $G_{K1}-V$ curve in Fig. S1). The AP morphology of the six cell models remains quite different, mainly because of the different degree of rectification (especially for Bett et al. (12)) and time dependency (Ishihara et al. (19) and O’Hara-Rudy (6)), not described by the steady-state I-V curves. On the other hand, the cell models that include *I*_*K*1_ according to Dhamoon et al. (18) and ten Tusscher (21) (both of them without time dependency) show similar behavior because they have comparable *G*_*K*1_ (see Fig. S1 *B*).

Upscaling *G*_*K*1_ stabilizes RMP toward potentials close to *E*_*K*_, speeds up the upstroke (higher ${\dot{V}}_{max}$), and hastens the repolarization (shorter *APD*_50_ and *APD*_90_). This behavior is coherent with Meijer van Putten et al. (27), who carried out DC experiments scaling the “electronic” *I*_*K*1_ density from 1 to 10 pA/pF. The identification of *G*_*K*1, *critical*_ represents the first step during DC experiments and, using the same *I*_*K*1_ formulation, allows comparison of cells under investigation (because they are in the same state), potentially reducing the intrinsic variability between cells.

The intrinsic variability among cells was computationally investigated by tailoring the hiPSC-CM Paci model in a cell-specific way, according to Lei et al. (24). Simulations highlighted that variations in *G*_*K*1, *critical*_ between the cell-specific models are small compared to the variation observed between the six different *I*_*K*1_ formulations tested. Using *G*_*K*1, *critical*_ to tune DC experiments will lead to a stable and hyperpolarized RMP with reduced variability between cells. Variability of APD and AP waveforms showed in Lei et al. (24) is preserved, also after addition of *I*_*K*1_ as in our simulations. Variability within the same cell line was shown experimentally by Verkerk et al. (11), studying hiPSC-CMs with and without *I*_*K*1_ addition with DC.

The APD observed in the cell-specific models showed median values comparable to the APD observed by Britton et al. (29) in human adult ventricular trabeculae under control conditions (≃300 ms; see Fig. 2 in (29)) and by O’Hara-Rudy et al. (6), who studied small epicardial tissues (≃280 ms; see Fig. 7 in (6)). Except for the models including the Bett *I*_*K*1_ formulation, all showed a median *APD*_90_ value that was higher than the mean value reported by Britton et al. (29) and O’Hara-Rudy et al. (6). Therefore, upscaling relative to *G*_*K*1, *critical*_ may be a strategy to make the repolarization faster and to close the gap between in silico and experimental data. Upscaling *G*_*K*1_ in a range between one and two times the identified critical value brings the AP features close to the experimental range, also in the initial Paci model.

The cell-specific models required slightly lower *G*_*K*1, *critical*_ values to stop the spontaneous activity than the initial Paci model. A difference between the identified values may be explained by the variability between AP waveform in the cell-specific models.

### Which *I*_*K*1_ formulation should be used in DC experiments?

The aim of adding *I*_*K*1_ to hiPSC-CMs using the DC technique is to bring about an electrophysiological phenotype that is as close to that of human adult CMs as possible because this will improve their predictive qualities in drug safety testing. The hyper-/depolarizing current prepulse protocol exposed the time-dependent properties of the *I*_*K*1_ formulation under investigation. The *I*_*K*1_ model based on experimental data from undiseased adult human ventricular cardiomyocytes by O’Hara-Rudy et al. (6) includes an instantaneous rectification factor $\left(R_{K1,\infty }\right)$ and an inactivation gating variable $\left(x_{K1,\infty }\right)$, described by a first-order kinetics. Despite the theoretical time dependency, the hiPSC-CM model including the O’Hara-Rudy *I*_*K*1_ formulation shows a near perfect overlap between the steady-state IV curve and the *I*_*K*1_ phase plots in all the tested conditions (control, hyperpolarizing, and depolarizing prepulse). Closer inspection of the simulation results demonstrated very minimal variation in *x*_*K*1_. This behavior can be explained by the voltage dependence of the $x_{K1,\infty }$ parameter for potentials in the AP range. Indeed, for *V*_*m*_ > −90 mV, the inactivation variables saturate to 1 (as also described in the original study by O’Hara-Rudy et al. (6); Fig. 2 *B*). The minimal variation in the inactivation parameters makes the time dependency of the O’Hara-Rudy *I*_*K*1_ formulation almost negligible (see Fig. S4) and explains the overlapping of the IV curves.

In contrast, the *I*_*K*1_ phase plots derived from the simulations using the Ishihara model show clear differences with the *I*_*K*1_ steady-state I-V curve. The three *I*_*K*1_ phase plots show a substantial current at more positive potentials because of the presence of a transient component. The transient component is determined by the influence of ${\left[Mg^{2+}\right]}_{i}$, polyamines (spermine $\left({\left[SPM\right]}_{i}\right)$, and spermidine $\left({\left[SPD\right]}_{i}\right)$) present in cardiomyocytes. The *I*_*K*1_ phase plots show various amounts of transient *I*_*K*1_ due to the different degree of relief of Mg^2+^ block. The hyperpolarizing prepulse, preceding the pacing stimulus, opens more channels, which can later become blocked by Mg^2+^ ions at depolarized potentials. The *I*_*K*1_ channels blocked at depolarized potentials become again available during repolarization, providing a stronger *I*_*K*1_ that shortens the APD. Vice versa, a depolarized prepulse reduces the amount of channels available for the binding with Mg^2+^ ions, leading to a weaker *I*_*K*1_ during repolarization and thus a prolonged APD, as previously shown by Ishihara et al. (19) employing the comprehensive guinea pig cell model (Kyoto model; see Fig. 4 *C* in Ishihara et al. (19)). The transient component of *I*_*K*1_ is an important aspect of *I*_*K*1_ because it can contribute to variation in APD and therefore proarrhythmia.

### Toward a mature electrophysiological phenotype

The third pillar of the CiPA initiative proposes to employ in vitro hiPSC-CMs to confirm the effects of a novel drug predicted by a comprehensive in silico model. hiPSC-CMs and adult ventricular CMs show qualitatively consistent responses to some, but not all drugs (9, 20, 30). Recently, two computational studies (8, 31) quantitatively reported on the main electrophysiological differences between hiPSC-CM and adult ventricular CMs by comparing the Paci 2013 and O’Hara-Rudy models. Paci et al. (22) investigated the discrepancies between hiPSC-CM and adult ventricular CMs by simulating the effects of pharmaceutical block of several membrane currents. They observed that the most relevant differences emerged during the block of $I_{Ca,L}$ and *I*_*K*1_ because of the overexpression of *I*_*NaCa*_ and a reduced repolarization reserve in hiPSC-CM. Gong and Sobie (31) systematically investigated the differences between hiPSC-CM and adult ventricular CM models and designed a mathematical approach to predict the effect of a drug on adult CMs based on recordings from hiPSC-CMs exposed to the drug. This approach is highly accurate when using in silico models; however, its in vitro validation is not easy to achieve: the availability of adult ventricular cell is scarce, and the variability between hiPSC-CM is likely to decrease the accuracy.

hiPSC-CMs with a mature electrophysiological phenotype are not available yet. Approaches such as increasing *I*_*K*1_ via dynamic clamping or ectopic overexpression of the KCNJ2 bring the AP waveform closer to that of an adult human cardiomyocyte. Combining this technique with the mathematical methods to extrapolate findings to adult human cardiomyocytes (such as proposed by Gong and Sobie (31)) may bring us closer to a predictive human cardiomyocyte model.

## Conclusions

In this exploratory in silico study, we addressed the issue of the immature electrophysiological profile of hiPSC-CMs. From the simulations, it can be concluded that 1) the electronic expression of *I*_*K*1_ according to Ishihara et al. (19) is able to successfully stop the automaticity of hiPSC-CMs and shows time-dependent properties that may be important for the evaluation of drug safety, and 2) the definition of *G*_*K*1, *critical*_ allows researchers to tailor the amount of *I*_*K*1_ for each cell, reaching an RMP comparable to adult CMs. *G*_*K*1, *critical*_ will be a sensitive parameter during in vitro DC experiments; it will be a sort of a fingerprint that characterizes every cell under investigation. The automatic identification of *G*_*K*1, *critical*_ will further facilitate implementation of DC on multichannel automated patch-clamp platforms, overcoming the low throughput that characterizes the combined use of manual patch clamping and DC.

### Limitations

The in silico results we reported in our work are based on the hiPSC-CM model published by Paci et al. in 2013 (22). More recently, updated or adapted versions of this model were published (32, 33). Adoption of these two new hiPSC-CM computational models is likely to lead to slightly different values for *G*_*K*1, *critical*_ when comparing the six *I*_*K*1_ formulations. In this study, we addressed potential hiPSC-CM model dependency of our conclusions by building cell-specific models based on the Paci 2013 model and the approach by Lei et al. (24). Indeed, the identified *G*_*K*1, *critical*_ values were slightly different but still very comparable to the values found using the initial Paci 2013 model. Kernik et al. (34) recently published their hiPSC-CM model based on several experimental data sets, collected in different laboratories. Because the model development is different with respect to the aforementioned models, the identification of $G_{K1,critical}$ in that model would bring further information about model dependency and cellular variability. Furthermore, this study used pacing at 1 Hz, a decision that was informed by our earlier work using human stem-cell-derived cardiomyocytes (35, 36). Using lower or higher pacing frequencies may affect the observed *G*_*K*1, *critical*_ values.

## Author Contributions

A.F., T.A.B.v.V., M.A.V., and T.P.d.B designed the research. A.F., B.G., and T.P.d.B. performed the research. A.F., B.G., and T.P.d.B. analyzed the data. A.F., B.G., M.A.V., T.A.B.v.V., and T.P.d.B. wrote the manuscript.

## Supporting Material

Document S1. Figs. S1–S6 and Tables S1 and S2

Document S2. Article plus Supporting Material

## References

[1] Kola I., Landis J. (2004). Can the pharmaceutical industry reduce attrition rates?. Nat. Rev. Drug Discov.

[2] Ferri N., Siegl P., Benghozi R. (2013). Drug attrition during pre-clinical and clinical development: understanding and managing drug-induced cardiotoxicity. Pharmacol. Ther.

[3] Laverty H., Benson C., Valentin J. (2011). How can we improve our understanding of cardiovascular safety liabilities to develop safer medicines?. Br. J. Pharmacol.

[4] Colatsky T., Fermini B., Stockbridge N. (2016). The comprehensive in vitro proarrhythmia assay (CiPA) initiative - update on progress. J. Pharmacol. Toxicol. Methods.

[5] Gintant G., Sager P.T., Stockbridge N. (2016). Evolution of strategies to improve preclinical cardiac safety testing. Nat. Rev. Drug Discov.

[6] O’Hara T., Virág L., Rudy Y. (2011). Simulation of the undiseased human cardiac ventricular action potential: model formulation and experimental validation. PLoS Comput. Biol.

[7] Paci M., Passini E., Rodriguez B. (2017). Phenotypic variability in LQT3 human induced pluripotent stem cell-derived cardiomyocytes and their response to antiarrhythmic pharmacologic therapy: an in silico approach. Heart Rhythm.

[8] Paci M., Hyttinen J., Severi S. (2015). Human induced pluripotent stem cell-derived versus adult cardiomyocytes: an in silico electrophysiological study on effects of ionic current block. Br. J. Pharmacol.

[9] Goversen B., van der Heyden M.A.G., de Boer T.P. (2018). The immature electrophysiological phenotype of iPSC-CMs still hampers in vitro drug screening: special focus on I_K1_. Pharmacol. Ther.

[10] Meijer van Putten R.M., Mengarelli I., Wilders R. (2015). Ion channelopathies in human induced pluripotent stem cell derived cardiomyocytes: a dynamic clamp study with virtual IK1. Front. Physiol.

[11] Verkerk A.O., Veerman C.C., Wilders R. (2017). Patch-clamp recording from human induced pluripotent stem cell-derived cardiomyocytes: improving action potential characteristics through dynamic clamp. Int. J. Mol. Sci.

[12] Bett G.C., Kaplan A.D., Rasmusson R.L. (2013). Electronic “expression” of the inward rectifier in cardiocytes derived from human-induced pluripotent stem cells. Heart Rhythm.

[13] Vaidyanathan R., Markandeya Y.S., Eckhardt L.L. (2016). IK1-enhanced human-induced pluripotent stem cell-derived cardiomyocytes: an improved cardiomyocyte model to investigate inherited arrhythmia syndromes. Am. J. Physiol. Heart Circ. Physiol.

[14] Wilders R. (2006). Dynamic clamp: a powerful tool in cardiac electrophysiology. J. Physiol.

[15] Ortega F.A., Grandi E., Christini D.J. (2018). Applications of dynamic clamp to cardiac arrhythmia research: role in drug target discovery and safety pharmacology testing. Front. Physiol.

[16] Goversen B., Becker N., de Boer T.P. (2018). A hybrid model for safety pharmacology on an automated patch clamp platform: using dynamic clamp to join iPSC-derived cardiomyocytes and simulations of I_k1_ ion channels in real-time. Front. Physiol.

[17] Lu G., Horvath A., de Boer T.P. (2019). Introducing simulated IK1 into human iPSC-cardiomyocytes using dynamic clamp on an automated patch clamp system. Biophys. J.

[18] Dhamoon A.S., Pandit S.V., Anumonwo J.M. (2004). Unique Kir2.x properties determine regional and species differences in the cardiac inward rectifier K+ current. Circ. Res.

[19] Ishihara K., Sarai N., Matsuoka S. (2009). Role of Mg(2+) block of the inward rectifier K(+) current in cardiac repolarization reserve: a quantitative simulation. J. Mol. Cell. Cardiol.

[20] Ma J., Guo L., January C.T. (2011). High purity human-induced pluripotent stem cell-derived cardiomyocytes: electrophysiological properties of action potentials and ionic currents. Am. J. Physiol. Heart Circ. Physiol.

[21] ten Tusscher K.H., Noble D., Panfilov A.V. (2004). A model for human ventricular tissue. Am. J. Physiol. Heart Circ. Physiol.

[22] Paci M., Hyttinen J., Severi S. (2013). Computational models of ventricular- and atrial-like human induced pluripotent stem cell derived cardiomyocytes. Ann. Biomed. Eng.

[23] Ishihara K., Yan D.H., Ehara T. (2002). Inward rectifier K(+) current under physiological cytoplasmic conditions in Guinea-pig cardiac ventricular cells. J. Physiol.

[24] Lei C.L., Wang K., Polonchuk L. (2017). Tailoring mathematical models to stem-cell derived cardiomyocyte lines can improve predictions of drug-induced changes to their electrophysiology. Front. Physiol.

[25] Garny A., Hunter P.J. (2015). OpenCOR: a modular and interoperable approach to computational biology. Front. Physiol.

[26] Cooper J., Scharm M., Mirams G.R. (2016). The cardiac electrophysiology web lab. Biophys. J.

[27] Meijer van Putten R.M., Mengarelli I., Wilders R. (2015). Ion channelopathies in human induced pluripotent stem cell derived cardiomyocytes: a dynamic clamp study with virtual IK1. Front. Physiol.

[28] Rocchetti M., Sala L., Zaza A. (2017). Elucidating arrhythmogenic mechanisms of long-QT syndrome CALM1-F142L mutation in patient-specific induced pluripotent stem cell-derived cardiomyocytes. Cardiovasc. Res.

[29] Britton O.J., Abi-Gerges N., Rodriguez B. (2017). Quantitative comparison of effects of dofetilide, sotalol, quinidine, and verapamil between human *ex vivo* trabeculae and *in silico* ventricular models incorporating inter-individual action potential variability. Front. Physiol.

[30] Matsa E., Rajamohan D., Denning C. (2011). Drug evaluation in cardiomyocytes derived from human induced pluripotent stem cells carrying a long QT syndrome type 2 mutation. Eur. Heart J.

[31] Gong J.Q.X., Sobie E.A. (2018). Population-based mechanistic modeling allows for quantitative predictions of drug responses across cell types. NPJ Syst. Biol. Appl.

[32] Paci M., Pölönen R.P., Hyttinen J. (2018). Automatic optimization of an *in silico* model of human iPSC derived cardiomyocytes recapitulating calcium handling abnormalities. Front. Physiol.

[33] Koivumäki J.T., Naumenko N., Tavi P. (2018). Structural immaturity of human iPSC-derived cardiomyocytes: in silico investigation of effects on function and disease modeling. Front. Physiol.

[34] Kernik D.C., Morotti S., Clancy C.E. (2019). A computational model of induced pluripotent stem-cell derived cardiomyocytes incorporating experimental variability from multiple data sources. J. Physiol.

[35] Jonsson M.K., Duker G., van Veen T.A. (2010). Quantified proarrhythmic potential of selected human embryonic stem cell-derived cardiomyocytes. Stem Cell Res.

[36] Jonsson M.K., Vos M.A., van Veen T.A. (2012). Application of human stem cell-derived cardiomyocytes in safety pharmacology requires caution beyond hERG. J. Mol. Cell. Cardiol.

[37] Koumi S., Sato R., Hayakawa H. (1994). Modulation of the delayed rectifier K+ current by apamin in Guinea-pig heart. Eur. J. Pharmacol.

[38] Yan D.-H., Ishihara K. (2005). Two Kir2.1 channel populations with different sensitivities to Mg(2+) and polyamine block: a model for the cardiac strong inward rectifier K(+) channel. J. Physiol.

