# Supplementary material for: Required GK1 to Suppress Automaticity of iPSC-CMs Depends Strongly on IK1 Model Structure
Source: Biophys J. 2019 Sep 13;117(12):2303–15. doi: 10.1016/j.bpj.2019.08.040 (PMC6990378; doi:10.1016/j.bpj.2019.08.040)
Supplement: Document S1. Figs. S1–S6 and Tables S1 and S2 [file mmc1.pdf]

Biophysical Journal, Volume *117*

Supplemental Information

Required G_{K1} to Suppress Automaticity of iPSC-CMs Depends Strongly on I_{K1} Model Structure

Alan Fabbri, Birgit Goversen, Marc A. Vos, Toon A.B. van Veen, and Teun P. de Boer

$G_{K1,critical}$ required for suppression of automaticity of iPSC-CM in dynamic clamp experiments strongly depends on I_{K1} model formulation used

A. Fabbri, B. Goversen, M.A. Vos, T.A.B. van Veen, T.P. de Boer

Supplementary Material

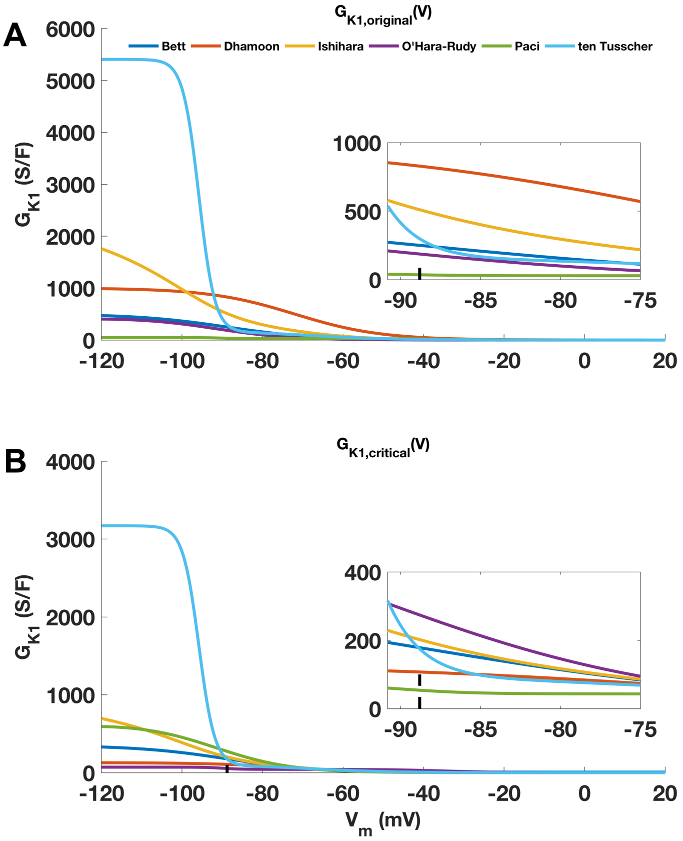

Figure S1: G_{K1}(V) in original (published) and critical conditions
A,B) G_{K1} depends on the membrane potential V_m. The insets magnify the G_{K1} in a voltage range close to the RMP. Larger G_{K1} is in this voltage range causes a hyperpolarized RMP, compare the insets and the table below.

$G_{K1,original}$		$G_{K1,critical}$	
**Model**	**RMP (mV)**	**Model**	**RMP (mV)**
Dhamoon	-88.1	O'Hara-Rudy	-86.8
Ishihara	-87.8	Ishihara	-85.9
Bett	-86.8	Bett	-85.9
ten Tusscher	-86.1	ten Tusscher	-83.7
O'Hara-Rudy	-	Dhamoon	-82.6
Paci	-77.2	Paci	-80.4

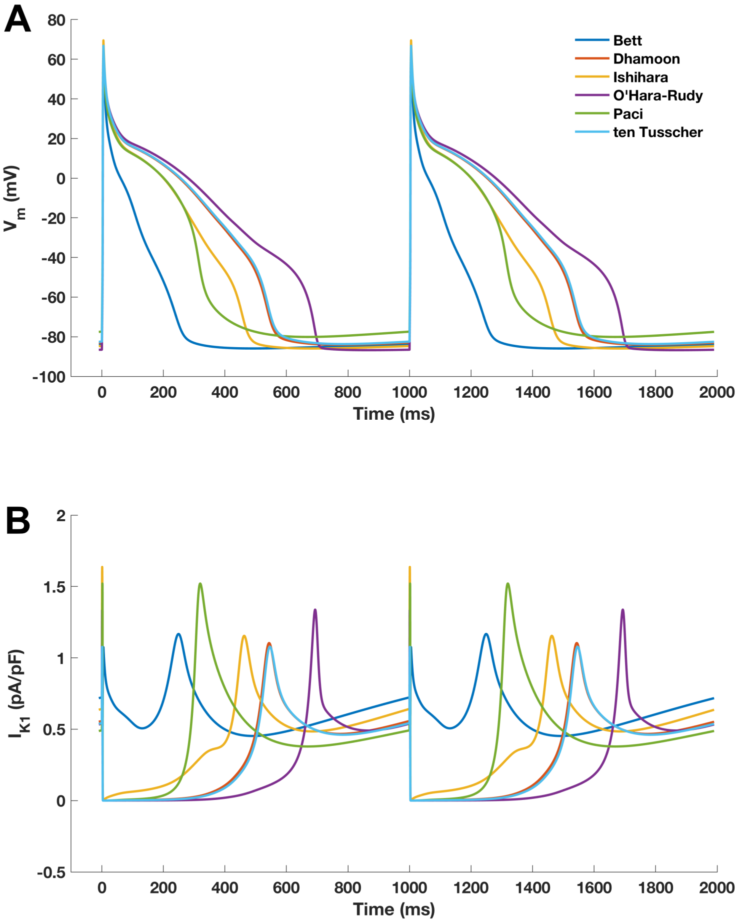

Figure S2: Comparison of I_{K1}formulations for G_{K1}=$G_{K1,critical}$

A) Action potential waveform and B) I_{K1} timecourse in the Paci hiPSC-CM models paced at 1Hz and with G_{K1}=$G_{K1,critical}$. Of note, (i) the models including the I_{K1}formulations according to Dhamoon and ten Tusscher (red and cyan traces) show nearly identical membrane potentials and I_{K1}. (ii) The low I_{K1}density during the diastolic interval is responsible for a less negative membrane potential in Paci

			Bett		Dhamoon		Ishihara		O'Hara-Rudy		Paci		ten Tusscher	
Cell ID	Scaling k NaCa	Scaling gKs	Critical GK1 (S/F)	IK1 avg (pA/pF)	Critical GK1 (S/F)	IK1 avg (pA/pF)	Critical GK1 (S/F)	IK1 avg (pA/pF)	Critical GK1 (S/F)	IK1 avg (pA/pF)	Critical GK1 (S/F)	IK1 avg (pA/pF)	Critical GK1 (S/F)	IK1 avg (pA/pF)
**1**	3.32	139	599.7	0.746	104.2	0.617	828.7	0.698	249.1	0.720	27.7	0.426	2500	0.590
**2**	2.08	129	597.4	0.717	104.2	0.593	818.1	0.670	249.2	0.695	27.9	0.414	2506	0.569
**3**	7.55	125	600.7	0.801	103.6	0.638	831.8	0.735	251.3	0.753	28.0	0.435	2465	0.602
**4**	21.1	467	597.5	0.916	101.1	0.742	824.9	0.882	253.9	0.988	0.0	0.000	2411	0.692
**5**	6.89	270	598.6	0.833	103.5	0.690	827.9	0.792	251.3	0.840	25.7	0.432	2470	0.651
**6**	9.3	211	598.9	0.843	103.6	0.686	830.3	0.793	252.6	0.838	26.3	0.435	2447	0.642
**7**	0.951	37.6	586.7	0.677	104.0	0.557	826.9	0.634	251.8	0.650	29.8	0.399	2513	0.535
**8**	1.54	19.5	590.7	0.684	104.5	0.528	828.6	0.610	252.8	0.597	29.2	0.391	2522	0.507
**9**	1.7	52	591.3	0.694	104.5	0.558	816.4	0.633	251.1	0.644	28.7	0.401	2516	0.535
**10**	1.67	0	590.9	0.680	104.6	0.480	835.6	0.578	252.9	0.511	29.3	0.379	2527	0.459
**11**	0.38	31.5	595.4	0.692	104.6	0.594	824.2	0.666	249.1	0.693	32.0	0.435	2539	0.573
**12**	0.696	18.6	590.2	0.676	104.1	0.558	829.7	0.635	251.0	0.645	30.4	0.401	2520	0.536
**13**	3.05	153	599.2	0.743	104.0	0.616	828.0	0.698	248.7	0.722	27.6	0.425	2498	0.590
**14**	1.28	125	589.0	0.693	104.0	0.582	808.6	0.654	249.9	0.688	28.7	0.407	2502	0.557
**15**	1.19	38.3	585.5	0.680	104.1	0.551	824.9	0.628	252.2	0.639	29.1	0.395	2515	0.529
**16**	3.02	47.3	600.0	0.718	104.9	0.563	823.0	0.641	249.8	0.637	28.2	0.405	2524	0.539
**17**	2.35	27	599.3	0.702	105.0	0.537	820.5	0.616	251.5	0.603	28.8	0.398	2528	0.514
**18**	4.03	138	600.4	0.760	104.3	0.626	828.8	0.709	249.7	0.730	27.5	0.427	2499	0.597
**19**	5.74	28.9	599.8	0.725	105.3	0.517	826.5	0.597	249.8	0.553	28.2	0.389	2545	0.494
**20**	2.35	9.18	598.5	0.695	105.0	0.502	827.5	0.591	252.8	0.547	29.0	0.387	2535	0.480
**21**	3.77	93.7	600.4	0.744	104.5	0.602	828.6	0.683	248.4	0.694	28.1	0.422	2507	0.575
**22**	13.9	288	599.8	0.883	102.3	0.714	819.9	0.833	251.7	0.904	0.0	0.000	2462	0.673
**Mean**			**595.9**	**0.741**	**104.1**	**0.593**	**825.4**	**0.681**	**250.9**	**0.695**	**25.9**	**0.373**	**2502**	**0.565**
**25th percentile**			591.0	0.692	104.0	0.553	823.3	0.629	249.7	0.638	27.6	0.392	2498	0.531
**median**			**598.6**	**0.717**	**104.2**	**0.587**	**827.2**	**0.660**	**251.2**	**0.690**	**28.2**	**0.403**	**2510**	**0.563**
**75th percentile**			599.7	0.756	104.6	0.623	828.7	0.706	252.1	0.728	29.1	0.425	2524	0.595
**min**			585.5	0.676	101.1	0.480	808.6	0.578	248.4	0.511	0.0	0.000	2411	0.459
**max**			600.7	0.916	105.3	0.742	835.6	0.882	253.9	0.988	32.0	0.435	2545	0.692

Table S1: Cell specific models: $G_{K1,critical}$ and average I_{K1} during late depolarization

We identified $G_{K1,critical}$ for each cell specific model (n=22) including the six I_{K1} formulations. The results of the bisection search are collected in the $G_{K1,critical}$ columns. I_{K1} avg represents the average I_{K1} flowing during the last 50 ms before the next stimulus when the cells are paced at 1 Hz.

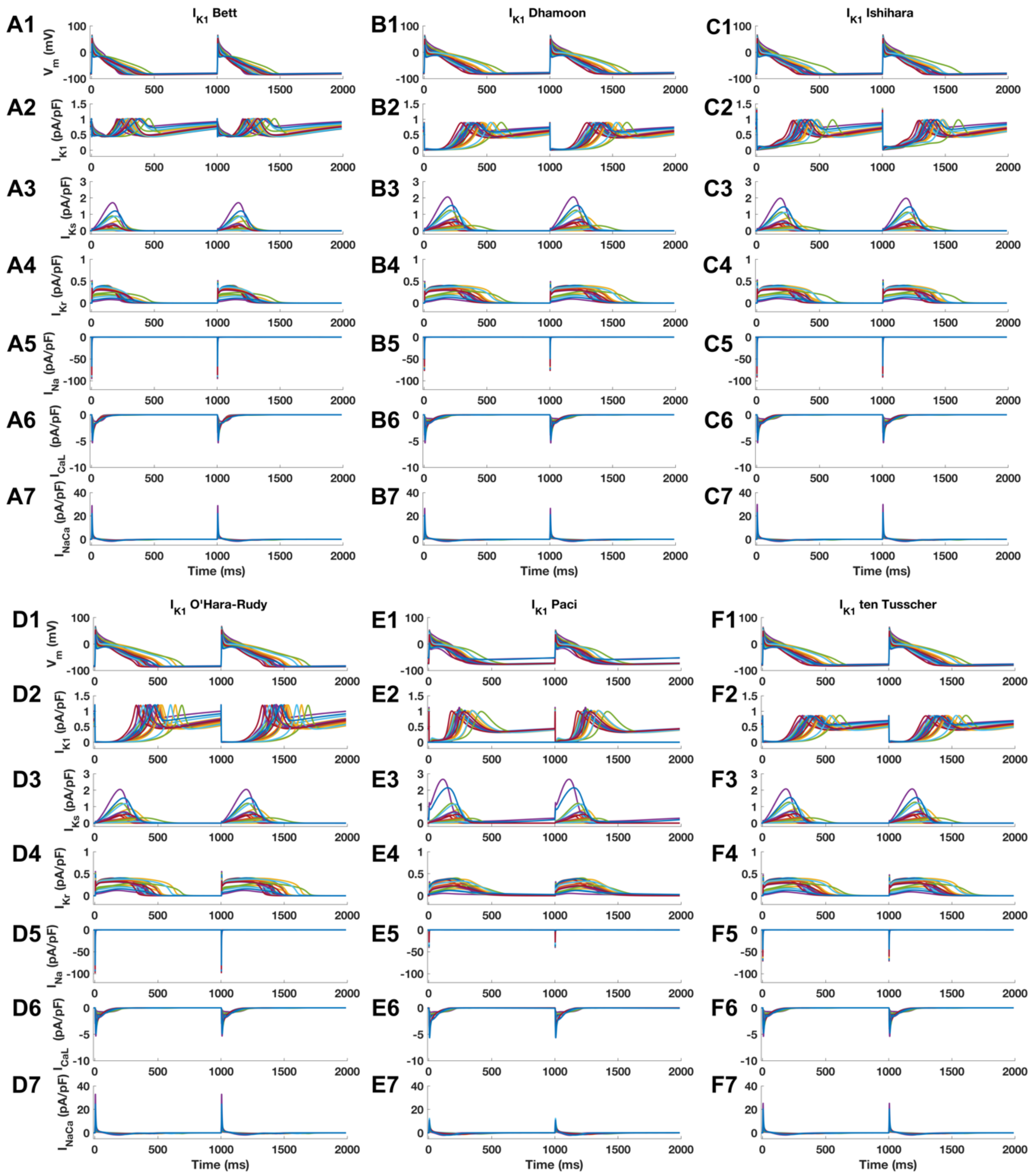

Figure S3: Membrane potential and main currents in the cell specific models

Membrane potential, I_{K1}, I_{Ks}, I_{Kr}, I_{Na} ,I_{CaL} and I_{NaCa} of the 22 cell specific models for each I_{K1} formulation. G_{K1} was set to the critical value identified running the bisection algorithm for each cell model

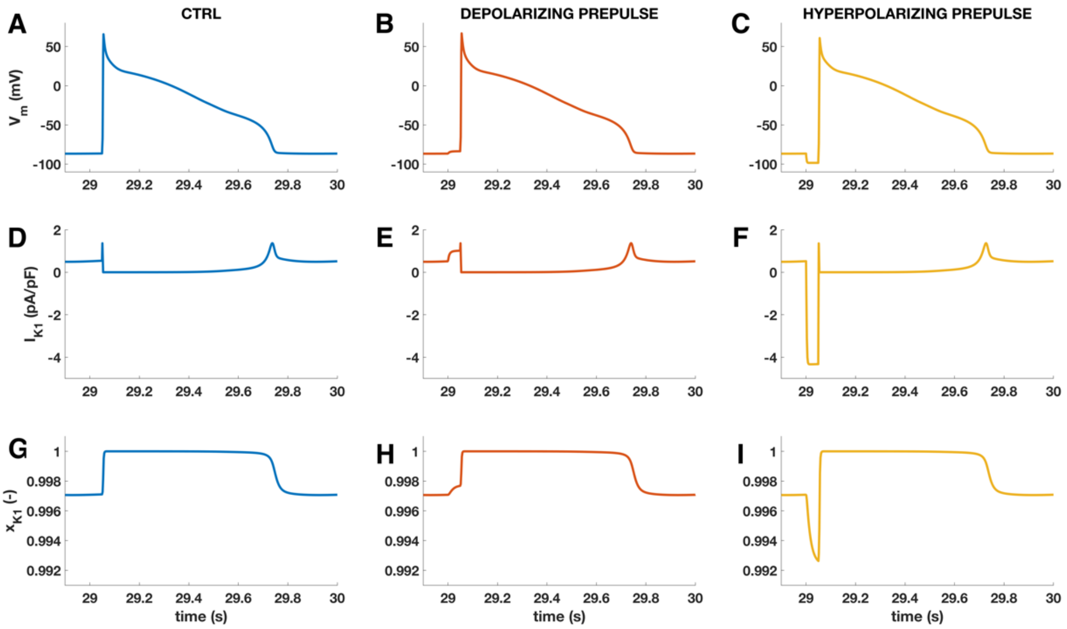

Figure S4: time dependency of I_{K1} in the O'Hara-Rudy formulation.
Membrane potential (A-C), I_{K1} density (D-F), and inactivation variable x_{K1} (G-I) time courses in CTRL conditions (first column) and in response to depolarizing and hyperpolarizing prepulses (second and third colums, respectively).
Panels G-I show the first order kinetics of x_{K1}, but since it's varying between 1 and 0.993, it can be considered negligible, even in CTRL conditions.

	Last APD_{90}				
**Model**	**CTRL**	**DEP**	**$\Delta APD_{90,Dep}$ (%)**	**HYPER**	**$\Delta APD_{90,Hyper}$ (%)**
**Bett**	237	240	1.3	232	-2.1
**Dhamoon**	540	544	0.7	522	-3.5
**Ishihara**	459	489	6.5	384	-16.3
**O'Hara-Rudy**	678	681	0.4	668	-1.5
**Paci**	356	357	0.3	339	-4.8
**ten Tusscher**	545	549	0.7	538	-1.3

Table S2: Preconditioning prepulse protocol
APD_{90} in the last AP of a cycle of thirty 1 Hz paced APs in CTRL conditions, in response to a depolarizing (DEP) or hyperpolarizing (HYPER) prepulse.

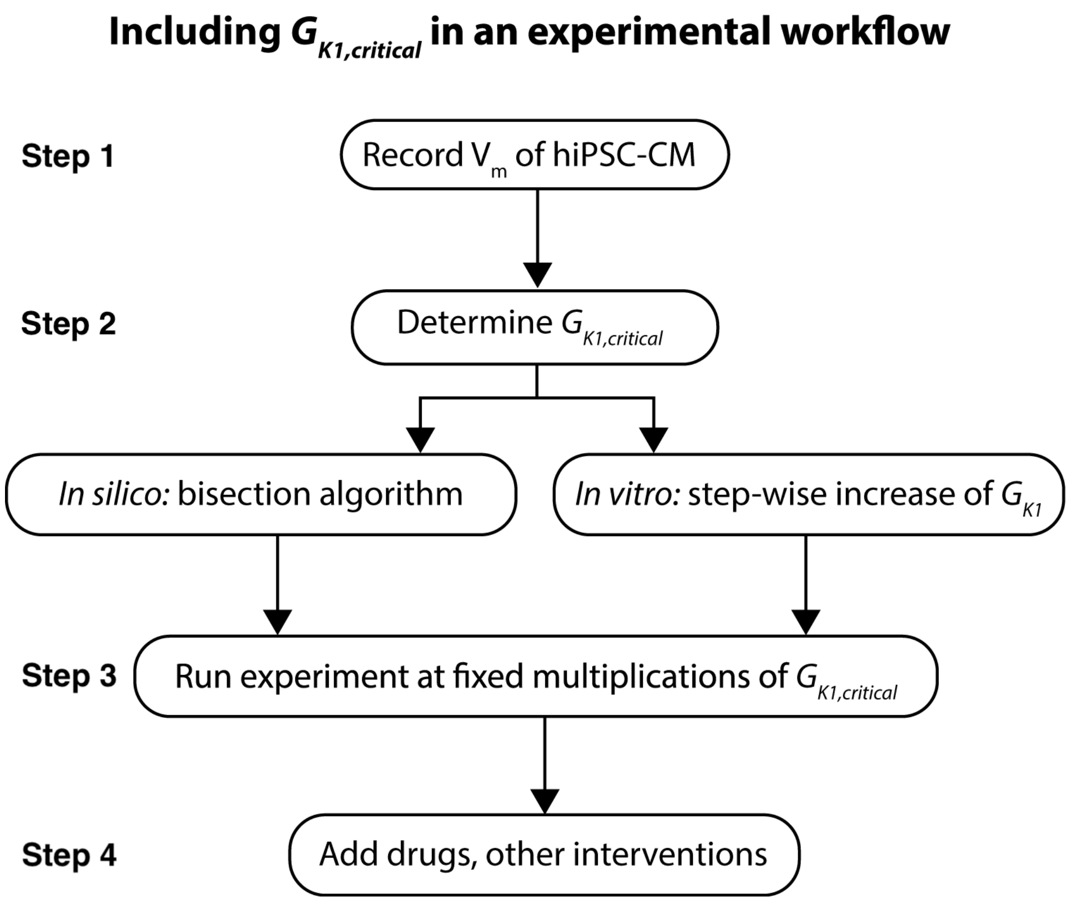

Figure S5: Including $G_{K1,critical}$ in an experimental workflow

Proposed steps in dynamic clamp or in silico experiments that include determination of $G_{K1,critical}$.

Step 1. Membrane potential is measured from round shaped and spontaneously beating hiPSC-CMs.

Step 2. *In silico* simulations allow to identify precisely $G_{K1,critical}$ for a cell specific or an average cell model. In an *in* vitro experiment, the bisection algorithm would take too long, and $G_{K1,critical}$ can instead be estimated with a protocol testing at step-wise increasing or decreasing G_{K1} values.

Step 3. Running subsequent experiments at multiplication of $G_{K1,critical}$ helps to reduce variation between cells.

Step 4. Measuring the AP while a drug is administered gives information about the efficacy or safety of the compound under investigation. Combination with results from a computational model could provide a mechanistic interpretation of what is observed during *in vitro* experiments

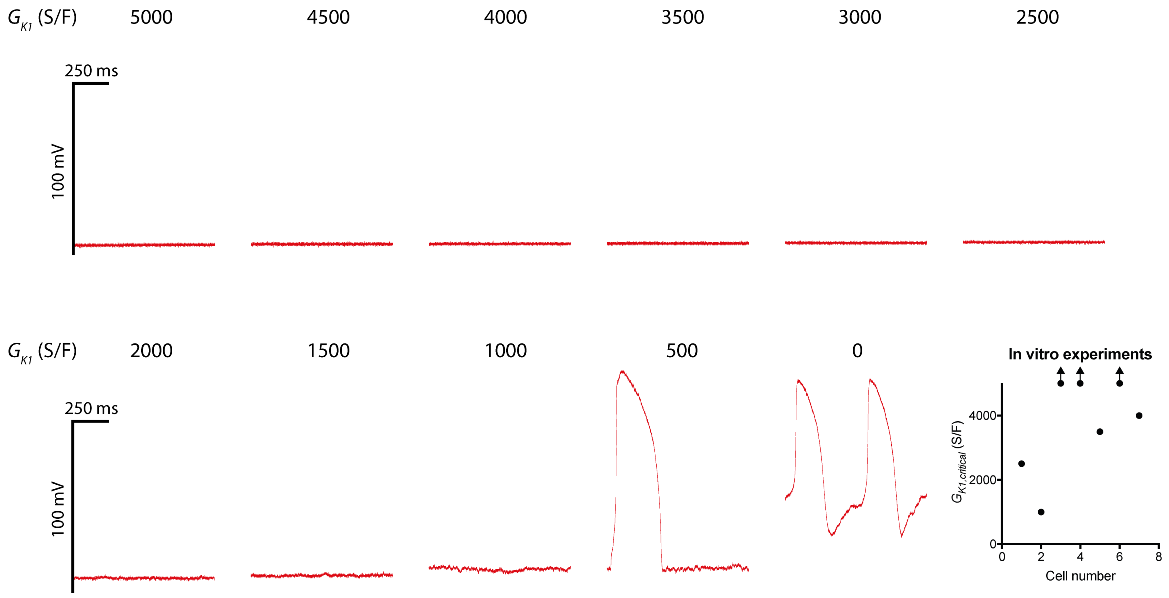

Figure S6: Determining $G_{K1,critical}$ in a dynamic clamp experiment

Main figure: In a set of dynamic clamp experiments using the Ishihara formulation, I_{K1} was injected using G_{K1} values starting at 5000 S/F, which was decreased with 500 S/F steps to 0 S/F. $G_{K1,critical}$ was defined as the minimal G_{K1} required for maintaining quiescence, in this example (Cell 2 in inset) $G_{K1,critical}$ was 1000 S/F. *Inset:* Summary plot of $G_{K1,critical}$ obtained in 7 cells. In the cells indicated with the arrows, the maximum G_{K1} tested did not completely suppress all activity, i.e. $G_{K1,critical}$ was slightly higher. The average $G_{K1,critical}$ in the 4 cells in which we obtained quiescence was 2750 ± 660 S/F.
