# Supplementary material for: Required GK1 to Suppress Automaticity of iPSC-CMs Depends Strongly on IK1 Model Structure
Source: Biophys J. 2019 Sep 13;117(12):2303–15. doi: 10.1016/j.bpj.2019.08.040 (PMC6990378; doi:10.1016/j.bpj.2019.08.040)
Supplement: Document S2. Article plus Supporting Material [file mmc2.pdf]

Biophysical Journal
Article

Biophysical Society

Required G_{K1} to Suppress Automaticity of iPSC-CMs Depends Strongly on I_{K1} Model Structure

Alan Fabbri,[1] Birgit Goversen,[1] Marc A. Vos,[1] Toon A. B. van Veen,[1] and Teun P. de Boer[1,*]
[1]University Medical Center Utrecht, Utrecht, the Netherlands

ABSTRACT Human-induced pluripotent stem cells derived cardiomyocytes (hiPSC-CMs) are a virtually endless source of human cardiomyocytes that may become a great tool for safety pharmacology; however, their electrical phenotype is immature: they show spontaneous action potentials (APs) and an unstable and depolarized resting membrane potential (RMP) because of lack of I_{K1}. Such immaturity hampers their application in assessing drug safety. The electronic overexpression of I_{K1} (e.g., through the dynamic clamp (DC) technique) is an option to overcome this deficit. In this computational study, we aim to estimate how much I_{K1} is needed to bring hiPSC-CMs to a stable and hyperpolarized RMP and which mathematical description of I_{K1} is most suitable for DC experiments. We compared five mature I_{K1} formulations (Bett, Dhamoon, Ishihara, O'Hara-Rudy, and ten Tusscher) with the native one (Paci), evaluating the main properties (outward peak, degree of rectification), and we quantified their effects on AP features (RMP, $\dot{V}_{max}$, APD_{50}, APD_{90} (AP duration at 50 and 90% of repolarization), and APD_{50}/APD_{90}) after including them in the hiPSC-CM mathematical model by Paci. Then, we automatically identified the critical conductance for I_{K1} ($G_{K1,\ critical}$), the minimally required amount of I_{K1} suppressing spontaneous activity. Preconditioning the cell model with depolarizing/hyperpolarizing prepulses allowed us to highlight time dependency of the I_{K1} formulations. Simulations showed that inclusion of mature I_{K1} formulations resulted in hyperpolarized RMP and higher $\dot{V}_{max}$, and observed $G_{K1,\ critical}$ and the effect on AP duration strongly depended on I_{K1} formulation. Finally, the Ishihara I_{K1} led to shorter (−16.3%) and prolonged (+6.5%) APD_{90} in response to hyperpolarizing and depolarizing prepulses, respectively, whereas other models showed negligible effects. Fine-tuning of G_{K1} is an important step in DC experiments. Our computational work proposes a procedure to automatically identify how much I_{K1} current is required to inject to stop the spontaneous activity and suggests the use of the Ishihara I_{K1} model to perform DC experiments in hiPSC-CMs.

SIGNIFICANCE In this work, we aim to contribute a method that will facilitate automated dynamic clamp (DC) experiments in which I_{K1} is injected in induced pluripotent stem-cell-derived cardiomyocytes (iPSC-CMs). By introducing $G_{K1,\ critical}$ (minimal I_{K1} conductance needed to stop automaticity of iPSC-CMs), we are proposing a different approach to setting up DC experiments. These are usually based on the injection of a fixed current density. In contrast, $G_{K1,\ critical}$ is a parameter that depends on the cell under investigation. Our in silico approach analyzed analogies and differences between I_{K1} formulations without the confounding factor that can be brought by the variability of iPSC-CMs. It highlighted how much the employed mathematical formulation of I_{K1} can affect $G_{K1,\ critical}$ and the action potential waveform in DC experiments.

INTRODUCTION

To be successfully placed on the market, a drug must be effective (i.e., it must be able to hit the desired target at clinically relevant concentrations) and safe (i.e., no side effects that could compromise the function of organs should occur). Insufficient efficacy and safety are responsible for almost 60% of the attrition rate in drug discovery and development (1,2). In the cardiovascular area, the proarrhythmic potential of a drug is the side effect reporting the highest number of postapproval adverse events (2,3).

Torsades de pointes is a potentially fatal arrhythmia that may occur when the repolarization of the ventricles is delayed. At the cellular scale, block of the human ether-à-go-go-related gene channels conducting the rapid delayed rectifier potassium current (I_{Kr}) is among the ones responsible for the delayed repolarization of the ventricles, which is detectable in surface electrocardiogram traces as a prolonged QT interval.

The International Committee on Harmonization S7b (preclinical) and the E14 (clinical) guidelines successfully

Submitted March 21, 2019, and accepted for publication August 26, 2019.
*Correspondence: t.p.deboer@umcutrecht.nl
Editor: Daniel Beard.
https://doi.org/10.1016/j.bpj.2019.08.040
© 2019 Biophysical Society.
This is an open access article under the CC BY license (http://creativecommons.org/licenses/by/4.0/).

reduced the risk of approving drugs that have the potential to induce torsades de pointes by deprioritizing development of drugs that block human ether-à-go-go related gene channels or cause QT prolongation. However, the guidelines are affected by low specificity (4). To overcome this problem, the comprehensive in vitro proarrhythmic assay (CiPA) initiative suggests an alternative paradigm to assess the safety of a new compound in the preclinical and early clinical stages (4,5). The paradigm shift consists of the hybrid combination of in vitro and in silico human-specific models (5). The first step in the CiPA paradigm focuses on the evaluation of the functional drug effects on inward currents involved in the plateau phase, such as the long-lasting calcium current (I_{CaL}) and the late sodium current ($I_{Na,L}$), and outward currents active during repolarization, such as the slow delayed rectifier potassium current (I_{Ks}), the inward rectifier potassium current (I_{K1}), and the transient outward potassium current (I_{to}). At the second stage, the experimental data are included into an in silico human adult ventricular action potential (AP) model (6) that allows one to investigate the behavior of the affected currents in an integrated environment. Finally, the prediction of the drug's effects is confirmed or disproved through in vitro experiments using human-induced pluripotent stem cells (hiPSCs) derived cardiomyocytes (hiPSC-CMs). Furthermore, in silico hiPSC-CM models have been successfully employed to assess drug efficacy. Paci et al. (7) showed that mexiletine and ranolazine, two multichannel blockers that target I_{Na}, I_{NaL}, I_{CaL}, and I_{Kr}, are able to recover the AP duration (APD) to physiological values in an LQTS3 hiPSC-CM population. In silico models of hiPSC-CMs also help to quantitatively characterize the differences between hiPSC-CM and adult ventricular cardiomyocytes (7). Indeed, the simulation of the pharmacological block of ion currents showed that the two cells types respond in different ways (8), giving a quantitative description of such a difference.

hiPSC-CMs are a promising tool for drug efficacy and safety testing. Because hiPSCs are obtained by reprogramming adult somatic cells to a pluripotent state, they are a virtually limitless source of cells with the same genome as the donor. Their origin from human adult cells avoids ethical issues associated with the use of human embryonic stem cells, bridge the gap resulting from the use of animal models, and bring personalized medicine closer. However, hiPSC-CMs are characterized by an immature electrophysiological phenotype that limits their employment in drug safety assessment (9). Unlike adult ventricular cardiomyocytes, they show high levels of expression of the HCN gene family (that encodes for the pacemaker current I_f) and low levels of the KCNJ2 gene, responsible for I_{K1}. The balance between ion channel types expressed by iPSC-CMs results in a depolarized resting membrane potential (RMP), a slow maximal upstroke velocity ($\dot{V}_{max}$) due to the inactivation of sodium channels, and the absence of the plateau phase. Furthermore, because of the contribution of I_f, hiPSC-CMs show spontaneous automaticity (9).

Several studies (10–12) have demonstrated that compensating for the low levels of I_{K1} improves the electrophysiological phenotype of hiPSC-CMs, making its phenotype more mature and more relevant for drug safety testing. Apart from attempts to improve differentiation protocols, two direct methods have been used to increase functional expression of I_{K1} in hiPSC-CMs (e.g., through adenoviral overexpression of I_{K1} channels (13) or by applying the dynamic clamp (DC) technique to insert virtual I_{K1} conductance) (14,15). The DC is a refinement of the traditional patch clamp. It allows one to interface the membrane potential of (one or more) cells with a computer running a real-time simulation of ion channels or gap junctions. In this way, it is possible to create a virtual electrical connection between cells or add a virtual ion channel to the cell membrane through a computational model that describes the time course of that current in response to membrane potential of the connected cell(s). The injected current can be fully described by mathematical equations and parameters. Through the tuning of the parameters, the amount of injected current can be modulated precisely to adapt it to the cell under investigation. The ability to fine-tune ion channel conductance to each cell being tested is essential when adding I_{K1} to hiPSC-CMs because too little will leave the cells beating spontaneously, whereas too much I_{K1} can render the cell nonexcitable. Earlier work by Verkerk et al. (11) indeed reported that the injection of I_{K1} eliminates the spontaneous activity of hiPSC and provides a more physiological phenotype (11). Bett et al. (12) highlighted that hiPSC-CMs respond like adult ventricular CMs to the administration of BayK-8644 only if I_{K1} is injected. Studies testing DC strategies to mature hiPSC-CMs have almost exclusively used manual patch clamping, but recently, our group successfully implemented DC-based I_{K1} injection in hiPSC-CM on an automatic patch-clamping platform that is capable of medium- to high-throughput drug screening by recording from up to eight cells in parallel (16,17).

Fine-tuning of the I_{K1} conductance (G_{K1}) is an important step in DC experiments, whether manual or automated patch clamping is used. Higher throughput applications however require an automated tuning procedure that can run unattended. In this study, we propose an approach to establish the required $G_{K1,\ critical}$ in a way that can be implemented on automated patch-clamping platforms. We define $G_{K1,critical}$ as the minimal value of conductance that is sufficient to bring the cell to a stable and hyperpolarized RMP. Identifying $G_{K1,\ critical}$ is relevant because it allows one to estimate the minimal amount of the injected I_{K1} for a cell, and it can subsequently be used as a reference value when G_{K1} is up- or downscaled, facilitating comparison of results obtained from different cells. Next to the value of G_{K1}, the time- and voltage-dependent properties of the used computational model of I_{K1} affect the AP waveform. Several

studies have captured the electrophysiology of I_{K1} in a computational model, providing descriptions with different levels of detail (6,12,18–21). The choice of the I_{K1} model used in DC experiments may therefore affect the resulting APs.

In this study, we have employed in silico DC experiments to explore the impact of I_{K1} on the immature hiPSC-CM electrophysiologic phenotype. Our simulations have the aim to 1) compare the effects on AP waveform of the six different I_{K1} formulations (6,12,18–21) in the hiPSC-CM computational model developed by Paci et al. (22), 2) establish an algorithm to estimate $G_{K1,\ critical}$ for the six models, 3) assess how the up- and downscaling of G_{K1} relative to $G_{K1,\ critical}$ affects the AP waveform, and 4) study the effects of hyper- or depolarizing prepulses on AP waveform.

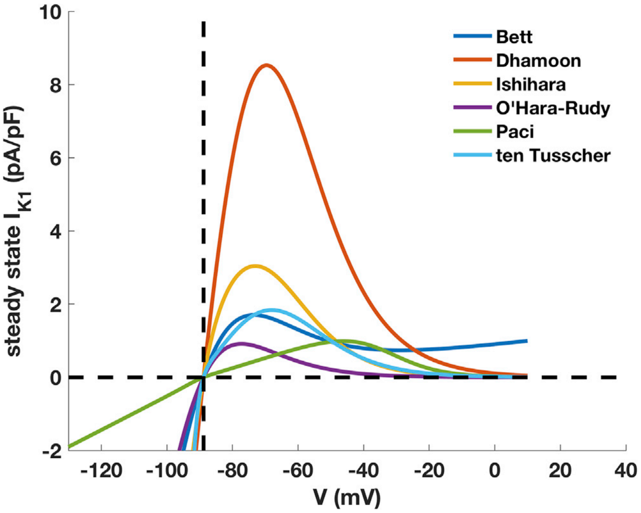

FIGURE 1 Comparison of the I_{K1} I-V curves of the six I_{K1} models for the original (published) G_{K1} value. E_K is marked by the vertical dashed line. To see this figure in color, go online.

MATERIALS AND METHODS

hiPSC-CM model, I_{K1} formulations and identification of $G_{K1,\ critical}$

We carried out the in silico experiments using the computational model of hiPSC-CM developed by Paci et al. (22). The Paci model is robustly constrained by experimental data obtained by Ma et al. (20), who electrophysiologically characterized hiPSC-CMs. Furthermore, they quantitatively investigate the mechanisms responsible for the immature electrophysiological phenotype, assessing the effects on spontaneous activity and AP waveform through the replacement of a subset of native currents (I_{Na}, I_{to}, $I_{Ca,L}$, I_{K1}, I_{Kr}, I_{Ks}) with adult ventricular current formulations from the O'Hara-Rudy model (6). The aforementioned characteristics make it a suitable model for evaluating DC strategies through adding I_{K1} conductances to hiPSC-CMs.

In this study, we have evaluated all I_{K1} model structures available in the literature, namely, those published by Bett et al. (12), Dhamoon et al. (18), Ishihara et al. (19), Paci et al. (22), O'Hara-Rudy et al. (6), and ten Tusscher et al. (21). The six I_{K1} formulations mainly differ in the presence (or absence) of time dependency in their kinetics and how the rectification of the outward current is described. A more detailed comparison between the six I_{K1} formulations is reported in Fig. 1 and Table 1. We set the extracellular potassium ion concentration $[K^+]_o$ to 5.4 mM and the intracellular concentration $[K^+]_i$ to 150 mM, setting the reversal potential for the six currents at $E_K = -88.8$ mV.

To study how the different formulations of I_{K1} affect the AP waveform, we paced hiPSC-CM cell models at 1 Hz, with a current stimulus of amplitude 15.2 pA/pF and 5 ms of duration, able to elicit AP in all the six models under comparison. The AP waveforms were quantitatively described through five biomarkers: the RMP, maximal speed of depolarization during the upstroke ($\dot{V}_{max}$), the APD at 50 and 90% of repolarization (APD_{50} and APD_{90}), and the ratio APD_{50}/APD_{90}, a biomarker to describe triangulation.

The identification of $G_{K1,\ critical}$ for each I_{K1} formulation was performed using the bisection algorithm in the unpaced hiPSC-CM. We performed the search in a range from 0 to 10 times the original (published) value. If the membrane potential of the cell was <−40 mV and with a difference between the minimal and maximal values over a period of 50 s that was <1 mV, the cell was defined as quiescent, and the current G_{K1} was stored as $G_{K1,high}$. On the contrary, if the cell showed spontaneous activity or failed to repolarize, G_{K1} was stored as $G_{K1,low}$; G_{K1} employed in the next step was calculated as $(G_{K1,high} + G_{K1,low})/2$. The G_{K1} value that made the cell quiescent and hyperpolarized was then challenged with a single external stimulus and the bisection search was carried out again. The algorithm stopped when the difference between $G_{K1,high}$ and $G_{K1,low}$ was lower than a tolerance set to 0.1% of the original value of G_{K1}.

As a proof of principle, in vitro experiments were carried out to determine $G_{K1,\ critical}$ in hiPSC-CM in a DC experiment using a custom DC system as described in Goversen et al. (16). Manual patch-clamp recordings were done at 37°C and followed the protocol outlined in Fig. S5. Coverslips with hiPSC-CM were superfused with a bath solution containing NaCl 130 mM, KCl 4 mM, $CaCl_2$ 1.8 mM, $MgCl_2$ 1.2 mM, $NaHCO_3$ 18 mM, HEPES 10 mM, and glucose 10 mM. Pipettes had resistances between 2 and 4 MΩ when filled with a pipette solution containing KCl 10 mM, K-gluconate 125 mM, $CaCl_2$ 0.6 mM, $MgCl_2$ 2 mM, HEPES 5 mM, sucrose 30 mM, Na_2 ATP 4 mM, and EGTA 5 mM. Liquid junction potential (+13.8 mV) was calculated using pCLAMP 10 and corrected a priori.

The effect of increasing or decreasing G_{K1} was investigated by running AP simulations paced at 1 Hz, using values for G_{K1} in a range from 0- to 10-fold $G_{K1,\ critical}$, increasing G_{K1} of $0.25 \times G_{K1,critical}$.

The last protocol aimed to reproduce the experiment by Ishihara et al. (23), using hyper-/depolarizing current prepulses. Before the pacing stimulus that triggered the AP, 50 ms current prepulses of 5 and −0.5 pA/pF were applied to respectively hyperpolarize or depolarize the voltage membrane. The combination of the prepulses and the pacing stimulus was administered at 1 Hz for 30 s.

Comparing the influence of cell-to-cell variation between iPSC-CMs and between I_{K1} formulations

The Paci 2013 model is based on the data collected by Ma et al. (20) using hiPSC-CMs. To address the variability between cells that is encountered in experiments, we identified $G_{K1,\ critical}$ for each I_{K1} formulation under investigation in a population of 22 cell-specific iPSC-CM models, published earlier by Lei et al. (24). In brief, Lei et al. (24) adapted the Paci 2013 model on the base of voltage clamp experiments carried out on hiPSC-CMs, by scaling the maximal conductance (S/F) of I_{Na} (×0.69), I_{CaL} (×0.80), I_{Ks}, and the maximal activity of I_{NaCa} (pA/pF) (tailored for each cell, the values are reported in Table 2). Next, we assessed the variability of the AP morphology while pacing the cell-specific models at 1 Hz.

Hardware and software

The Paci-based hiPSC-CM models including the six I_{K1} formulations were encoded in CellML and solved using OpenCOR (version 0.6) (25) and on

TABLE 1 Main Characteristics of the Six I_{K1} Formulations

I_{K1} Formulation	Experimental Data Source	Transient	Steady State	Rectification	Conductance	Reference
Bett	adult human ventricular cells	NP	steady state only	voltage dependent	constant	(12,37)
Dhamoon	guinea pig Kir2.1 in HEK293	NP	steady state only	voltage dependent	constant	(10,18)
Ishihara	mouse Kir2.1 in HEK 293	v	v	two channel populations sensitive to $[Mg^{2+}]_i$ and $[SPM]_i$	$[K^+]_o$ dependent	(19,38)
O'Hara-Rudy	adult human ventricular cells	inactivation gate	v	voltage dependent $[K^+]_o$ dependent	$[K^+]_o$ dependent	(6)
Paci	hiPS-CM	NP	steady state only	voltage dependent	$[K^+]_o$ dependent G_{K1} ×1.1	(20,22)
ten Tusscher	adult human ventricular and atrial	NP	steady state only	voltage dependent	$[K^+]_o$ dependent	(21,37)

NP, not present; v, implemented.

the Cardiac Electrophysiology Web Lab (available at https://travis.cs.ox.ac.uk/FunctionalCuration/) (26). Simulations done using OpenCOR were performed on a macOS High Sierra (10.13.6) Apple computer (Apple, Cupertino, CA) equipped with 2.9 GHz quad-core Intel Core i7 (Intel, Santa Clara, CA). We used a variable step method for stiff problems (backward differentiation formula) implemented in the CVODE library. The currents and the state variables of the models were stored and displayed with a 0.1 ms time step. The cell models achieved steady-state conditions when the difference of the norm of the state variable vector at the beginning and at the end of the AP was lower than 10^{-6}. The extraction of AP waveform features and plots were performed in MATLAB (release R2018a; The Mathworks, Natick, MA).

RESULTS

hiPSC-CM AP waveform is strongly influenced by I_{K1} model structure

A first comparison between the six I_{K1} formulations was made by plotting the steady-state current generated by the models at voltages between −120 and +10 mV (see Fig. 1). The Dhamoon model shows the highest outward peak (8.5 pA/pF at −69.5 mV), whereas I_{K1} from the O'Hara-Rudy model shows the lowest outward peak current density (0.91 pA/pF at −77.1 mV). The Paci model, constructed using experimental data obtained from immature hiPSC-CMs, is remarkably different from the other models, which are based on experimental data from adult myocytes or heterologous expression systems, and reaches the outward peak (0.99 pA/pF) at more depolarized potentials (−46.1 mV). The model structures also differ in the rectification: the Dhamoon model shows I_{K1} density close to zero at depolarized potentials ($V_m > 0$ mV), whereas the O'Hara-Rudy model already reaches small current densities around −40 mV. The Bett model differs markedly at positive potentials, showing a clear positive linear growth.

Next, we simulated paced APs using the Paci models incorporating the six I_{K1} models. Analysis of AP waveform allows quantification of the effects of the I_{K1} formulations. The cell models that include the Dhamoon and the Paci I_{K1} formulations show the most hyperpolarized and depolarized RMPs and the fastest and the lowest $\dot{V}_{max}$ (−88.1 mV, 160.9 V/s, and −77.2 mV, 57.9 V/s, respectively). The key role played by I_{K1} in repolarization becomes clear when comparing APD_{50} and APD_{90} values. On one hand, the activity of I_{K1} during the plateau and phase 3 of the Bett model leads to shorter AP durations (APD_{50} = 58 ms, APD_{90} = 153 ms). On the other hand, in the model including the O'Hara-Rudy formulation, the low amplitude of I_{K1} is not sufficient to successfully repolarize the membrane potential that settles to a stable but depolarized RMP (−20.4 mV), as also reported by Paci et al. (22).

Calculating the ratio of APD_{50} and APD_{90} (APD_{50}/APD_{90}) gives some additional insight about the repolarization course. Values close to 0.5 result from APs with a triangular shape for which the plateau phase is virtually absent and the repolarization is monotonic, whereas values close to 1 occur when phase 3 is steep, and thus the final phase of repolarization is fast. The Bett model shows a value (APD_{50}/APD_{90} = 0.38) that is coherent with a triangular waveform, whereas the Dhamoon model has APD_{50}/APD_{90} = 0.76, consistent with a steep phase 3 repolarization. The Ishihara, the Paci, and ten Tusscher models show $APD_{50}/APD_{90} > 0.5$ (0.57, 0.67, 0.60, respectively).

To get a better understanding of the influence of the six models on emerging AP waveforms, we compared the AP waveforms and underlying I_{K1} current profiles (see Fig. 2).

TABLE 2 AP Waveform Parameters of hiPSC-CM Models Including the six I_{K1} Formulations at $G_{K1,\ original}$

Model	RMP (mV)	$\dot{V}_{max}$ (V/s)	APD_{50} (ms)	APD_{90} (ms)	APD_{50}/APD_{90} (−)
Paci (unpaced)	−76.9	24.9	366	487	0.75
Bett	−86.8	157.5	58	153	0.38
Dhamoon	−88.1	160.9	101	133	0.76
Ishihara	−87.8	157.9	84	163	0.57
O'Hara-Rudy	−20.4	NA	NA	NA	NA
Paci	−77.2	57.9	302	453	0.67
ten Tusscher	−86.1	136.4	258	431	0.60

The APD_{50}/APD_{90} ratio provides a quantification of the shape of the AP waveform.

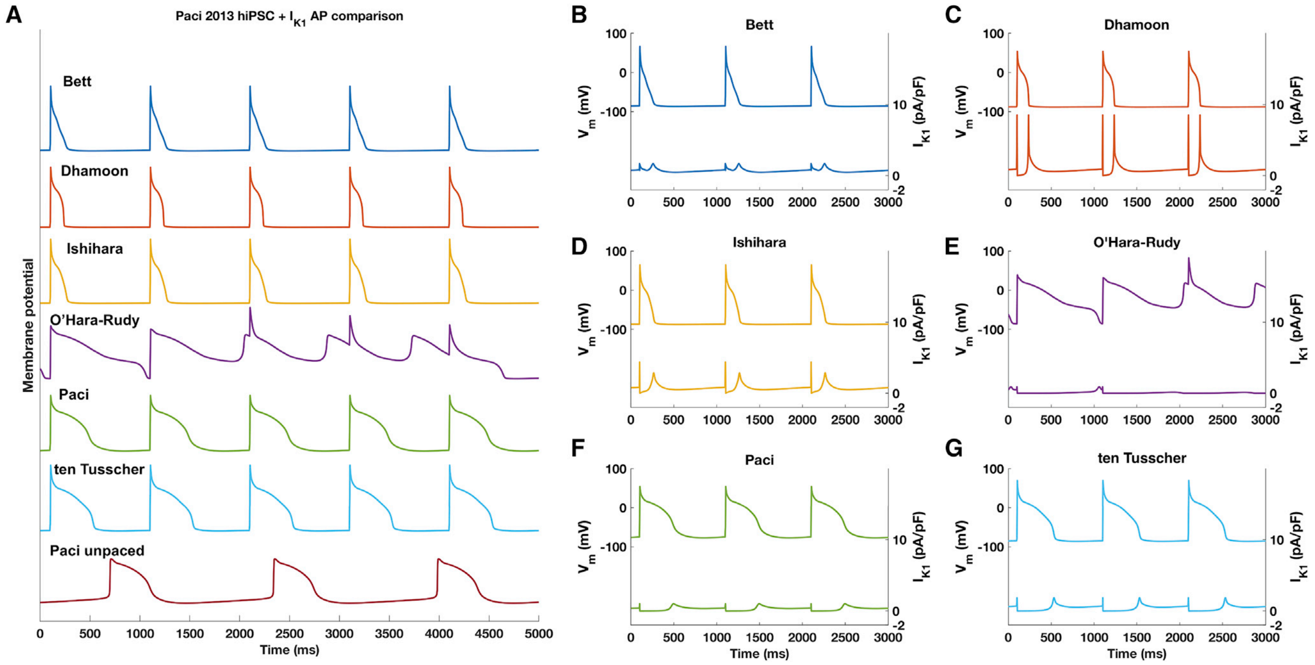

FIGURE 2 (*A*) AP waveforms elicited by pacing the Paci hiPSC-CM model incorporating the six different I_{K1} formulations at 1 Hz, with the original G_{K1}. The bottom trace displays the original hiPSC-CM Paci model without pacing, which shows the spontaneous activity that is typical of hiPSC-CMs. (*B–G*) V_m and I_{K1} time courses of the hiPSC-CM cell models including the six I_{K1} formulations are shown. The model including O'Hara-Rudy I_{K1} formulation failed to repolarize after 70 s; here, we report the transition from successful to failing repolarization (fourth trace in *A* and *E*). To see this figure in color, go online.

This showed a clear effect of I_{K1} model structure on the AP waveform. The Dhamoon I_{K1} shows the largest outward peak (8.5 pA/pF), as expected from the steady-state I-V characteristic. The Bett current is active throughout all AP phases but mostly during the plateau phase (~1 pA/pF), during which the other models produce currents of negligible amplitude. The Ishihara I_{K1} contributes to the early phase 3 of the AP, starting earlier during the repolarization phase than the Dhamoon, Paci, and ten Tusscher I_{K1} models. The Dhamoon model shows the largest peak (8.5 pA/pF) during late repolarization at which it contributes to a fast final repolarization toward the RMP. The Dhamoon and the Ishihara models both bring the RMP (−88.1 and −87.8 mV, respectively) close to E_K (−88.8 mV) by conducting a current density of ~0.8 pA/pF. The lowest I_{K1} density during phase 4 is generated by the Paci model, which also shows the least hyperpolarized RMP (−77.2 mV).

Scaling relative to $G_{K1,\ critical}$ facilitates comparison of I_{K1} models or cells

The critical value of G_{K1} ($G_{K1,\ critical}$) is an estimation of the minimal number of I_{K1} channels that is required on the cell membrane to suppress automaticity, to make the cell 1) quiescent, 2) with an RMP that is stable and close to E_K, even when the cell is perturbed with an external stimulus. After establishing $G_{K1,\ critical}$ for all six I_{K1} models, we observed two different behaviors. When incorporating the Bett, Dhamoon, Ishihara, or ten Tusscher models, the hiPSC-CM models become quiescent with G_{K1} values that are lower than those in the original I_{K1} models (see Table 3), with a decrease of

TABLE 3 AP Features of the 1 Hz Paced hiPSC-CM Models for $G_{K1} = G_{K1,\ critical}$

Model	$G_{K1,\ original}$ (S/F)	$G_{K1,\ critical}$ (S/F)	ΔG_{K1} (%)	RMP (mV)	$\dot{V}_{max}$ (V/s)	APD_{50} (ms)	APD_{90} (ms)	APD_{50}/APD_{90} (−)
Bett	1000	685.8	−31.4	−85.9	145.0	85	242	0.35
Dhamoon	1000	129.4	−87	−84.0	112.8	301	541	0.56
Ishihara	2500	989.7	−60.4	−85.9	132.3	238	458	0.52
O'Hara-Rudy	190.8	279.8	+46.6	−86.7	112.8	349	686	0.51
Paci	28.149	43.3	+53.8	−80.1	85.1	242	367	0.66
ten Tusscher	5405	3170	−41.3	−83.7	108.2	306	546	0.56

ΔG_{K1}, variation (in %) between the original and critical value of G_{K1}; $G_{K1,\ critical}$, critical value of Kir2.1 conductance that brings RMP to stable and hyperpolarized values; $G_{K1,\ original}$, default value of Kir2.1 conductance.

31.4, 87, 60.4, and 41.3%, respectively. On the other hand, the Paci and O'Hara-Rudy models needed an increase of G_{K1} (+53.8 and +46.6%, respectively) to stabilize RMP; this means that these two models do not provide enough I_{K1} in their original formulation to stop the automaticity. Time course of membrane potential and IK1 are depicted in Fig. S2.

Fig. 3, *A–F* depicts the AP waveform with G_{K1} values ranging between 0 and 10× $G_{K1,\ critical}$. The downscaling of G_{K1} resulted in longer APDs and a depolarized RMP when the cells were able to repolarize. Progressively increasing G_{K1} resulted in a faster repolarization process with a smaller APD_{50} and APD_{90} (Fig. 3, *G* and *H*). A higher amount of I_{K1} is also responsible for a hyperpolarized and stable RMP (Fig. 3 *I*) that gets close to E_K. The inset in Fig. 3 *I* shows that for a conductance corresponding to $G_{K1,\ critical}$, RMPs are still quite different: the hiPSC-CM model including the Paci formulation is the most depolarized (−80.1 mV), whereas the model including the O'Hara-Rudy formulation is the most hyperpolarized (−86.7 mV).

For all I_{K1} models, the upstroke velocity $\dot{V}_{max}$ (Fig. 3 *J*) increased with increasing G_{K1} values from ~0.5 to 2× $G_{K1,\ critical}$ and then stabilizes at values around 150 V/s for larger G_{K1} values. For G_{K1} > 7.5× $G_{K1,\ critical}$ and > 9.25× $G_{K1,\ critical}$, respectively, the pacing stimulus was no longer sufficient to trigger an AP when using the Ishihara or the O'Hara-Rudy models (Fig. 3, *C* and *D*, see *insets*). Together with $G_{K1,\ critical}$, this behavior suggests that there is a range for the amount of I_{K1} that should be injected into the cell, providing indications for in vitro experiments.

$G_{K1,\ critical}$ shows limited variability between cell-specific iPSC-CM models

Variation in AP waveform between hiPSC-CM is well known, and this may influence the $G_{K1,\ critical}$ value required in a DC experiment for maintaining quiescence of a particular cell. To test this, we employed 22 cell-specific iPSC-CM models, tailored on the Paci 2013 model, with variations in ion currents based on voltage clamp data (24). For each cell-specific model, we identified $G_{K1,\ critical}$ using the six I_{K1} formulations we wanted to assess. Fig. 4 illustrates the range of the 22 $G_{K1,\ critical}$ values obtained for each I_{K1} formulation. $G_{K1,\ critical}$ did not follow a normal distribution; therefore, we described them through the median and 25th and 75th percentiles. Median $G_{K1,\ critical}$ was 598.6 (I_{K1} according to Bett), 104.2 (Dhamoon), 827.2 (Ishihara),

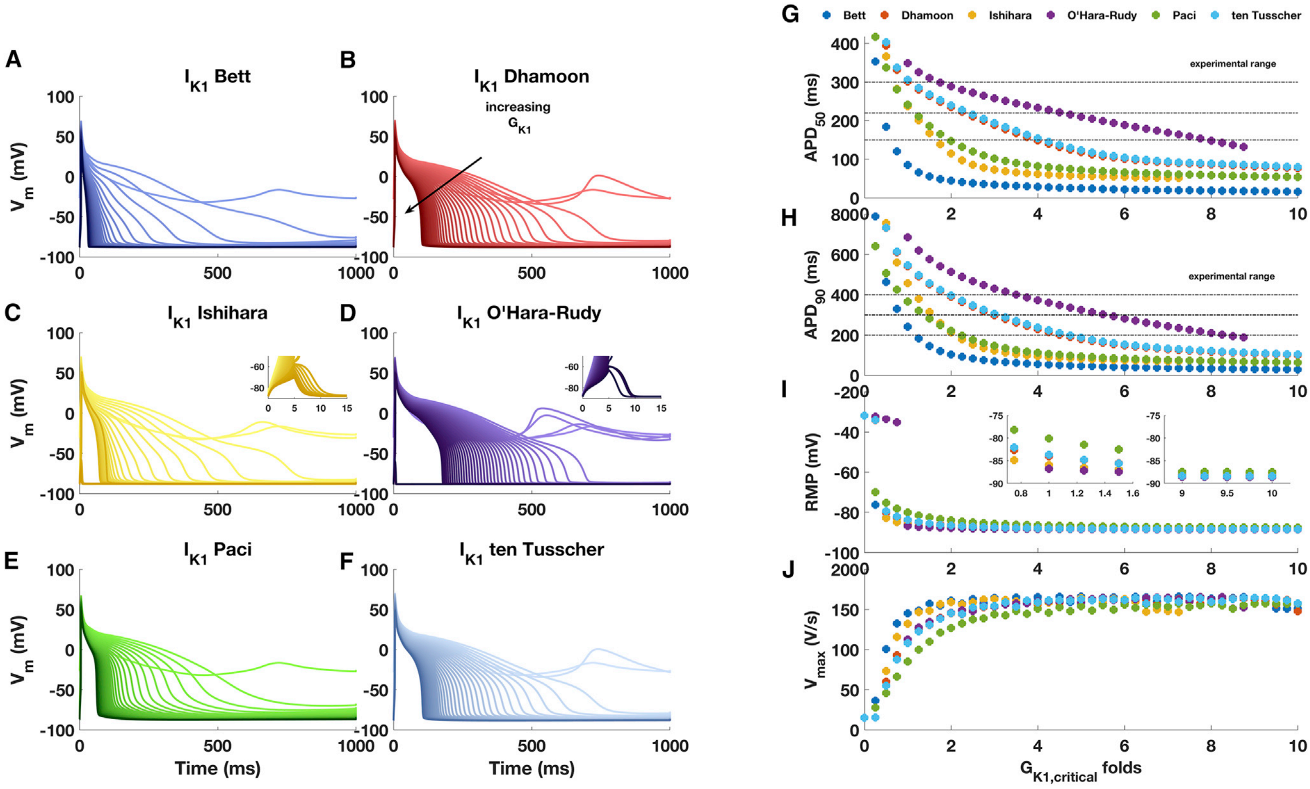

FIGURE 3 (*A–F*) AP waveforms at varying G_{K1} formulations. Note how a G_{K1} value that is too large prevents the pacing stimulus from triggering an AP for cell models that include Ishihara et al. (19) and O'Hara-Rudy et al. (6) I_{K1} (insets in *C* and *D*). Light colors code for low G_{K1} values, and dark colors code for high G_{K1} values. (*G–J*) AP features (APD_{50}, APD_{90}, RMP, and $\dot{V}_{max}$) extracted from the membrane potential time courses are shown. Simulations were run using G_{K1} values ranging from 0 (no I_{K1}) to 10×$G_{K1,critical}$ and increase with 0.25×$G_{K1,critical}$ step. The experimental range for APD_{50} and APD_{90} refers to adult healthy cardiomyocytes from Britton et al. (29). To see this figure in color, go online.

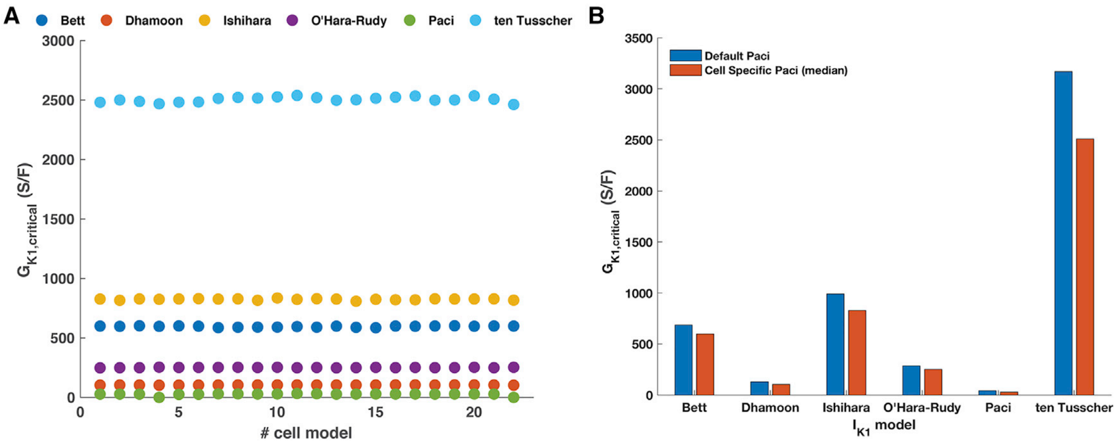

FIGURE 4 (*A*) $G_{K1,\ critical}$ values of each of the 22 cell specific Paci models, including the I_{K1} formulations according to Bett et al. (12), Dhamoon et al. (18), Ishihara et al. (19), Paci et al. (22), O'Hara-Rudy et al. (6), and ten Tusscher et al. (21). (*B*) Comparison between $G_{K1,\ critical}$ identified in the default Paci model (*blue bars, left*) and the median $G_{K1,\ critical}$ for the cell specific Paci models (*red bars, right*). Notably, $G_{K1,\ critical}$ varies more between I_{K1} formulations than between cells. To see this figure in color, go online.

28.2 (Paci), 251.2 (O'Hara-Rudy), and 2510 S/F (ten Tusscher). Within the six populations of cell-specific models, we found a limited range for $G_{K1,\ critical}$: the population including the I_{K1} according to Paci reported the largest variation of the 25th and 75th percentile from the median $G_{K1,\ critical}$ (−2.47 and +2.82%, respectively), whereas the models including the other I_{K1} formulations showed variations lower than 1%. Table S1 reports in detail the cell-specific $G_{K1,\ critical}$ identified for each cell.

To test if our findings with the cell-specific models were reflected in in vitro experiments, a small set of DC experiments was done using hiPSC-CM. We injected I_{K1} using the formulation by Ishihara and determined $G_{K1,\ critical}$ (see Figs. S5 and S6). In in silico experiments, conditions are ideal, and we were able to check if the membrane potential was stable and in steady state during a 50-s-long time window for many tested G_{K1} values. During in vitro experiments, membrane potential is less stable because of noise, and testing many 50-s iterations of the bisection algorithm takes more time than typically feasible in patch-clamp experiments. For the aforementioned reasons, we did not implement a bisection algorithm but instead started from a large G_{K1} value (5000 S/F), which was decreased in steps of 500 S/F. The average $G_{K1,\ critical}$ was 2750 ± 660 S/F ($n = 4$), with values ranging from 1000 to 4000 S/F, confirming the cell-specific nature of the $G_{K1,\ critical}$ parameter. In three additional cells, slightly more than 5000 S/F was needed to suppress minimally remaining automaticity, but the correct value could not be obtained before the experiment expired.

The comparison between the median $G_{K1,\ critical}$ found in the cell-specific models and the one identified in the initial models used earlier (Fig. 4 *B*) helps to get more insight in the differences between cells. In the cell-specific models, for all the included I_{K1} formulations, the $G_{K1,\ critical}$ value observed was lower than the one identified in the initial Paci 2013 models. The distance between the initial Paci 2013 and the cell-specific models was the highest in the models including I_{K1} according to Paci (−38.2%), whereas it reached −12.3% when the models included the O'Hara-Rudy I_{K1} formulation. The difference among $G_{K1,\ critical}$ in the other I_{K1} formulations was within the boundaries set by Paci and O'Hara-Rudy I_{K1} models.

We further assessed the behavior of the cell-specific models by eliciting APs with external stimuli at 1 Hz, using the cell-specific $G_{K1,\ critical}$. RMP was hyperpolarized, close to E_K and within a limited range (Fig. 5 *A*; Fig. S3), consistent with the low variability of $G_{K1,\ critical}$. I_{K1} contributes to stabilization of RMP. We compared the contribution of the I_{K1} formulations by measuring the average I_{K1} current density during the diastolic interval, just before the external stimulus (Fig. 5 *B*). The amount of current is comparable between the six I_{K1} formulations but consistent with the less negative RMP, the population that includes the Paci I_{K1} model reported a lower average I_{K1} density during the diastolic interval. APD_{50} and APD_{90} showed stronger variability (Fig. 5, *C* and *D*), which is consistent with the cell-specific variations in I_{Ks} and I_{NaCa} current densities. The population including I_{K1} according to Bett showed the shortest APD_{90} (287 ms), whereas the others are included within 300 and 450 ms. For a detailed overview of the AP waveform of the cell-specific models, see Fig. S3 and Table S1.

Reproducing the effect of prepulses on APD requires time dependence of I_{K1}

Although most I_{K1} models do not include a time constant in the equations describing rectification, experimental data have demonstrated an effect of time dependence of I_{K1} channel rectification (23), as brief prepulses given before the pacing stimulus have been shown to affect APD in guinea pig ventricular cardiomyocytes. Specifically, a hyperpolarizing

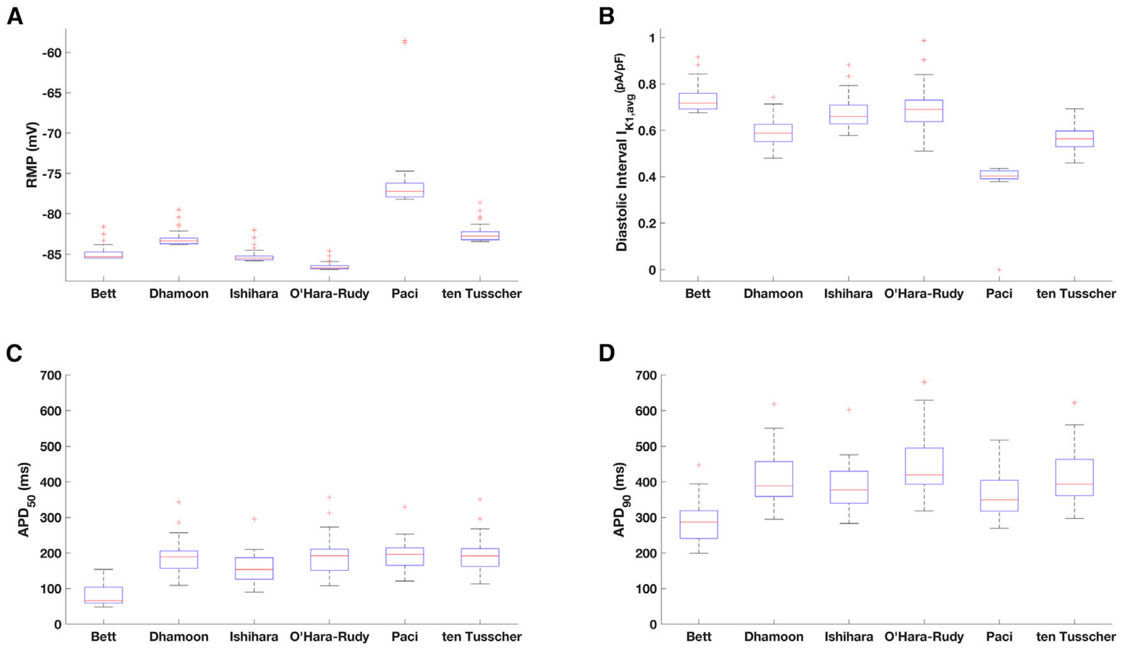

FIGURE 5 Main AP features of the cell-specific models paced at 1 Hz. (*A*) RMPs are within in a limited range. The population of models including I_{K1} according to Paci et al. (22) showed a less negative RMP ($RMP_{median} = -77.4$ mV). (*B*) The average I_{K1} density during the last 50 ms of the diastolic interval (before the stimulus) is shown; because of the less negative RMP, the current density is lower in the population that includes the Paci I_{K1}. (*C* and *D*) APD_{50} and APD_{90} show a higher variability within the populations. To see this figure in color, go online.

prepulse caused faster repolarization, that is, a shorter APD, whereas a depolarizing prepulse led to APD prolongation. It was demonstrated that I_{K1} was the current underlying this behavior because of the different availability of open channels at hyperpolarized/depolarized membrane potentials. We tested whether prepulses affect the APD in the initial hiPSC-CM models with the six different I_{K1} models because contribution of I_{K1} to variation in repolarization duration can affect the outcome of DC experiments aimed testing proarrhythmic properties of drugs. Fig. 6, *A*1–6 and *B*1–6 depict the AP waveforms and I_{K1} profiles of the last beat of a train of paces of the six hiPSC-CM models. At first sight, it is clear that the hiPSC-CM model including the Ishihara I_{K1} formulation is the only one to show significant effects on AP, as the hyperpolarizing current prepulse caused APD_{90} prolongation (+6.5%), whereas the depolarizing prepulse shortened APD_{90} (−16.3%) with respect to the AP elicited with no prestep, reproducing qualitatively the experimental data. The effects observed in the other AP models were negligible (see Table S2). For the O'Hara-Rudy model, this was surprising because this I_{K1} model does include a time constant in the description of rectification by including an instantaneous rectification factor ($R_{K1,\infty}$) and an inactivation gating variable ($x_{K1,\infty}$). In the simulations of hiPSC-CM with the O'Hara-Rudy I_{K1}, closer inspection of the behavior of these parameters showed that x_{K1} was only minimally affected by the prepulses, ranging between 1 and 0.993 (minimal value obtained with the hyperpolarizing prepulse; see Fig. S4, *G*–*I*). Fig. 6 *C*1–6 shows I_{K1} phase plots in which I_{K1} density is plotted against V_m during repolarization, similar to a phase diagram depicting $\dot{V}_m$ versus V_m. The Ishihara formulation provided different amounts of I_{K1} in response to the prepulses. In dynamic conditions, the I_{K1} phase plots are different from I_{K1} in steady state, showing a larger amount of I_{K1} available also at more depolarized potentials. In particular, when the cell is preconditioned with a hyperpolarizing prepulse, it is possible to notice a further smaller I_{K1} peak (0.66 pA/pF) at −38.5 mV responsible for the APD shortening. In contrast, no appreciable differences were observed with the other models in which the I_{K1} phase plots overlapped the steady-state IV curve. Therefore, the I_{K1} formulation that we consider the most suitable to implement in DC experiments is the one according to Ishihara et al. (19).

DISCUSSION

hiPSC-CMs are a promising tool for drug safety screening and to study cardiac arrhythmia mechanisms. They represent a pillar in the CiPA initiative and allow researchers to study the effects of drugs on AP morphology in an integrated cellular environment with similarities to human adult cardiomyocytes. The main characteristic that hampers their application in assessing drug safety is their spontaneous activity that leads to an unstable and depolarized RMP ($V_m > -60$ mV). Previous studies demonstrated that is possible to improve the "immature" phenotype of hiPSC-CM through the overexpression of I_{K1} either by virally overexpressing I_{K1} channels in the cells (13) or through "electronic expression" using the DC

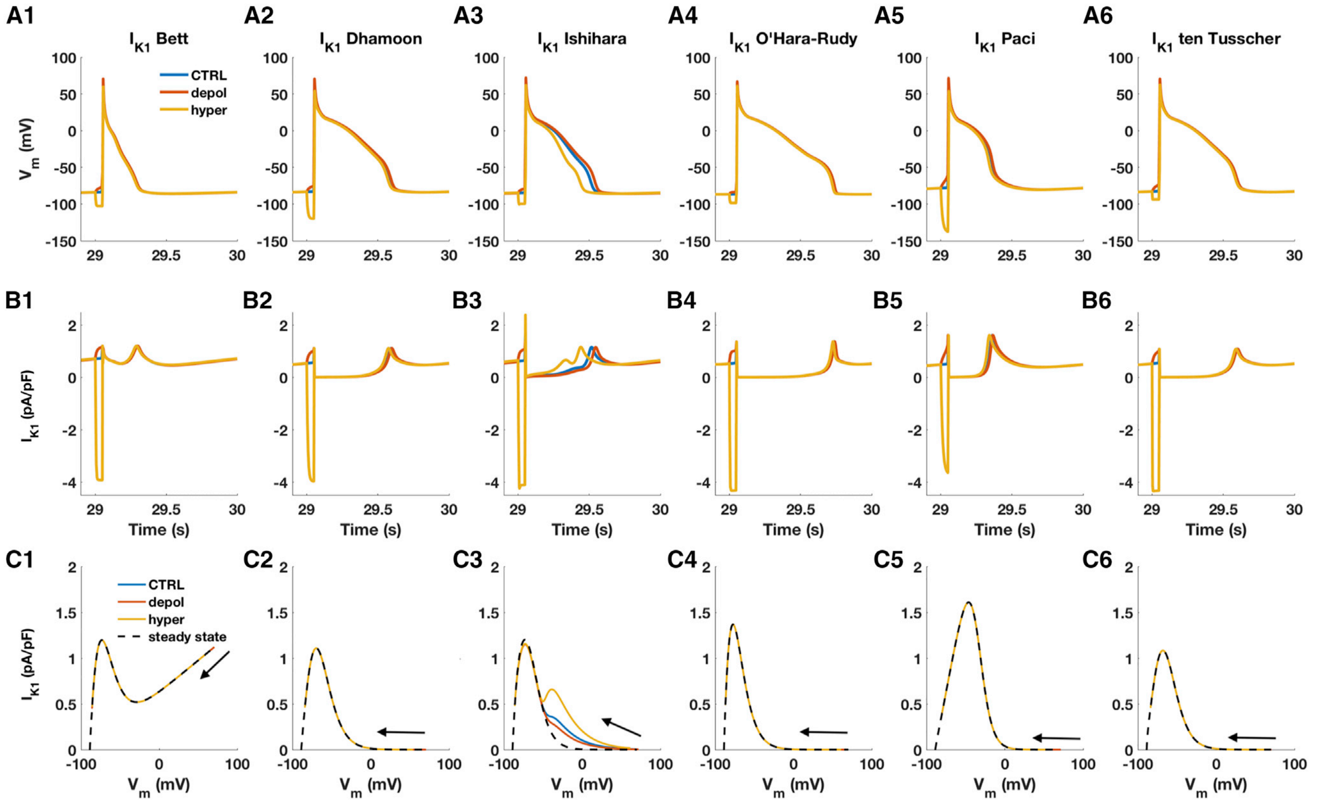

FIGURE 6 (A1–6 and B1–6) Membrane potential and I_{K1} waveforms for the hiPSC-CM models including the six I_{K1} formulations during the last beat. The cell models were paced at 1 Hz for 30 s. The hyperpolarizing and the depolarizing current prepulse had an amplitude of 5 and −0.5 pA/pF, respectively, and were applied for 50 ms before the AP triggering pulse. Note that only the model including the Ishihara I_{K1} formulation shows significant effects on AP duration (APD). (C1–6) I_{K1} phase plots of I_{K1} plotted against membrane potential during repolarization (*blue*, *red*, and *yellow solid lines*) and in steady state (*black dashed lines*) are shown. Arrows point from the start of repolarization toward the end. The Ishihara et al. (19) I_{K1} formulation provides an outward current at more depolarized potential during the repolarization phase. Such outward current contribution is not present in steady-state conditions in which I_{K1} density is much smaller at depolarized potentials. No differences between dynamic IV curves during repolarization and during voltage clamp protocol (steady-state conditions) were observed in the other models. To see this figure in color, go online.

technique (11,12,16,27). DC is a powerful technique because it is able to mimic I_{K1} carried by biological ion channels. Although it is not able to reproduce the molecular interactions that real I_{K1} channels can participate in, DC does allow fine control of the amount of I_{K1} added and allows the experimentalists to change it "on the fly," adapting it to characteristics (e.g., maturity, AP waveform, cell size) of the cell under investigation.

Our study aims to answer two main questions. 1) How much I_{K1} has to be injected to stop the automaticity with a stable RMP? 2) Which is the most suitable I_{K1} mathematical formulation to employ during in vitro DC experiments? We addressed these two questions through a computational approach, using the Paci et al. model (22) as a virtual hiPSC-CM and simulating DC experiments. To this end, we 1) compared a subset of I_{K1} formulations present in literature and assessed how they affect the AP waveform of the simulated hiPSC-CM, 2) identified the minimal amount of virtual I_{K1} channels needed to stop the automaticity through the parameter $G_{K1,critical}$, 3) investigated the effects on AP waveform features of down-/up-scaling of I_{K1}, and 4) tested the capability of the I_{K1} formulations to reproduce experimental data reporting APD dependency on hyper- or depolarizing prepulses through an I_{K1}-specific mechanism.

I_{K1} formulation and its effects on AP waveform

The comparison of the steady-state I-V curves highlights the differences between the I_{K1} formulations under investigation. The original Paci et al. (22) model includes I_{K1}, characterized by a low outward peak current at depolarized potentials (∼−46 mV). The other I_{K1} models derived from adult cardiomyocyte models show substantial differences in the outward peak current or in the degree of rectification. The peak outward current density ranges between 1 (O'Hara-Rudy et al. (6)) and ∼8 pA/pF (Dhamoon et al. (18)). The I_{K1} formulation according to Bett et al. (12) shows a low degree of rectification, with substantial I_{K1} density also at more depolarized potentials. The Ishihara et al. (19) I_{K1} formulation has a more complex mathematical formulation compared to the other models and quantitatively describes how the

block exerted by intracellular cations as Mg^{2+} and polyamines can affect the ion channel gating.

In silico experiments allowed us to assess the effects of the aforementioned properties on the AP waveform, avoiding disturbances caused by the high variability of AP waveform in real hiPSC-CMs (11). When the evaluated I_{K1} formulations replace the native one in the Paci et al. (22) model, RMP becomes stable and hyperpolarized. This holds true for models with all tested I_{K1} formulations when paced at 1 Hz, but the I_{K1} formulated according to O'Hara-Rudy et al. (6) leads to stable but depolarized RMP (−20.4 mV), as showed by Paci et al. (22). These results highlight that the amount of I_{K1} provided by the O'Hara-Rudy et al. (6) model in the original formulation is not enough to stabilize RMP to hyperpolarized values. Simulations confirmed the link between hyperpolarized RMP and $\dot{V}_{max}$; the overexpression of I_{K1} brings the cell to a different working point (i.e., the gating variables are in a different state), unveiling I_{Na} that is responsible for a faster upstroke. A strong I_{K1} is responsible for a faster repolarization (see the steep phase 3 in the hiPSC-CM model including the formulation according to Dhamoon et al. (18)), and this characteristic, together with a strong rectification, leads to APD_{50}/APD_{90} ratios >0.5. On the other hand, the low degree of rectification in the Bett et al. (12) formulation is responsible for a substantial current (∼1 pA/pF) at depolarized potentials that abolished the plateau phase. Our results are in agreement with the study by Verkerk et al. (11) that included in the Paci et al. (22) model the I_{K1} formulations according to Meijer van Putten et al. (27), Bett et al. (12), and Rocchetti et al. (28). In their simulations, they reported a stable and hyperpolarized RMP, a faster $\dot{V}_{max}$, and a faster repolarization. As reported in our simulation, they also showed that Bett et al. (12) I_{K1} dramatically shortened the *APD*.

How much I_{K1} should be injected to suppress spontaneous activity?

Earlier studies employing DC to inject I_{K1} in hiPSC-CMs have typically standardized the amount of added I_{K1} relative to cell capacitance (e.g., a fixed peak outward current density). In this study, we have described an alternative approach based on the critical G_{K1}, defined as the parameter that depicts the minimal number of I_{K1} channels on the cell membrane needed to bring the membrane potential to hyperpolarized and stable values, thereby suppressing the spontaneous activity. Our DC experiments in hiPSC-CM indeed confirm that different cells have a different $G_{K1,\ critical}$ value (Fig. S6).

In our simulations, the identification of $G_{K1,\ critical}$ for each model showed a substantial decrease (up to −87%) for the I_{K1} formulations according to Bett et al. (12), Dhamoon et al. (18), Ishihara et al. (19), and ten Tusscher et al. (21). AP waveform analysis of the simulated AP highlights the correlation between G_{K1} and RMP: higher G_{K1} leads to more hyperpolarized RMP (see also the $G_{K1} - V$ curve in Fig. S1). The AP morphology of the six cell models remains quite different, mainly because of the different degree of rectification (especially for Bett et al. (12)) and time dependency (Ishihara et al. (19) and O'Hara-Rudy (6)), not described by the steady-state I-V curves. On the other hand, the cell models that include I_{K1} according to Dhamoon et al. (18) and ten Tusscher (21) (both of them without time dependency) show similar behavior because they have comparable G_{K1} (see Fig. S1 *B*).

Upscaling G_{K1} stabilizes RMP toward potentials close to E_K, speeds up the upstroke (higher $\dot{V}_{max}$), and hastens the repolarization (shorter APD_{50} and APD_{90}). This behavior is coherent with Meijer van Putten et al. (27), who carried out DC experiments scaling the "electronic" I_{K1} density from 1 to 10 pA/pF. The identification of $G_{K1,\ critical}$ represents the first step during DC experiments and, using the same I_{K1} formulation, allows comparison of cells under investigation (because they are in the same state), potentially reducing the intrinsic variability between cells.

The intrinsic variability among cells was computationally investigated by tailoring the hiPSC-CM Paci model in a cell-specific way, according to Lei et al. (24). Simulations highlighted that variations in $G_{K1,\ critical}$ between the cell-specific models are small compared to the variation observed between the six different I_{K1} formulations tested. Using $G_{K1,\ critical}$ to tune DC experiments will lead to a stable and hyperpolarized RMP with reduced variability between cells. Variability of APD and AP waveforms showed in Lei et al. (24) is preserved, also after addition of I_{K1} as in our simulations. Variability within the same cell line was shown experimentally by Verkerk et al. (11), studying hiPSC-CMs with and without I_{K1} addition with DC.

The APD observed in the cell-specific models showed median values comparable to the APD observed by Britton et al. (29) in human adult ventricular trabeculae under control conditions (≃300 ms; see Fig. 2 in (29)) and by O'Hara-Rudy et al. (6), who studied small epicardial tissues (≃280 ms; see Fig. 7 in (6)). Except for the models including the Bett I_{K1} formulation, all showed a median APD_{90} value that was higher than the mean value reported by Britton et al. (29) and O'Hara-Rudy et al. (6). Therefore, upscaling relative to $G_{K1,\ critical}$ may be a strategy to make the repolarization faster and to close the gap between in silico and experimental data. Upscaling G_{K1} in a range between one and two times the identified critical value brings the AP features close to the experimental range, also in the initial Paci model.

The cell-specific models required slightly lower $G_{K1,\ critical}$ values to stop the spontaneous activity than the initial Paci model. A difference between the identified values may be explained by the variability between AP waveform in the cell-specific models.

Which I_{K1} formulation should be used in DC experiments?

The aim of adding I_{K1} to hiPSC-CMs using the DC technique is to bring about an electrophysiological phenotype

that is as close to that of human adult CMs as possible because this will improve their predictive qualities in drug safety testing. The hyper-/depolarizing current prepulse protocol exposed the time-dependent properties of the I_{K1} formulation under investigation. The I_{K1} model based on experimental data from undiseased adult human ventricular cardiomyocytes by O'Hara-Rudy et al. (6) includes an instantaneous rectification factor ($R_{K1,\infty}$) and an inactivation gating variable ($x_{K1,\infty}$), described by a first-order kinetics. Despite the theoretical time dependency, the hiPSC-CM model including the O'Hara-Rudy I_{K1} formulation shows a near perfect overlap between the steady-state IV curve and the I_{K1} phase plots in all the tested conditions (control, hyperpolarizing, and depolarizing prepulse). Closer inspection of the simulation results demonstrated very minimal variation in x_{K1}. This behavior can be explained by the voltage dependence of the $x_{K1,\infty}$ parameter for potentials in the AP range. Indeed, for $V_m > -90$ mV, the inactivation variables saturate to 1 (as also described in the original study by O'Hara-Rudy et al. (6); Fig. 2 *B*). The minimal variation in the inactivation parameters makes the time dependency of the O'Hara-Rudy I_{K1} formulation almost negligible (see Fig. S4) and explains the overlapping of the IV curves.

In contrast, the I_{K1} phase plots derived from the simulations using the Ishihara model show clear differences with the I_{K1} steady-state I-V curve. The three I_{K1} phase plots show a substantial current at more positive potentials because of the presence of a transient component. The transient component is determined by the influence of $[Mg^{2+}]_i$, polyamines (spermine ($[SPM]_i$), and spermidine ($[SPD]_i$)) present in cardiomyocytes. The I_{K1} phase plots show various amounts of transient I_{K1} due to the different degree of relief of Mg^{2+} block. The hyperpolarizing prepulse, preceding the pacing stimulus, opens more channels, which can later become blocked by Mg^{2+} ions at depolarized potentials. The I_{K1} channels blocked at depolarized potentials become again available during repolarization, providing a stronger I_{K1} that shortens the APD. Vice versa, a depolarized prepulse reduces the amount of channels available for the binding with Mg^{2+} ions, leading to a weaker I_{K1} during repolarization and thus a prolonged APD, as previously shown by Ishihara et al. (19) employing the comprehensive guinea pig cell model (Kyoto model; see Fig. 4 *C* in Ishihara et al. (19)). The transient component of I_{K1} is an important aspect of I_{K1} because it can contribute to variation in APD and therefore proarrhythmia.

Toward a mature electrophysiological phenotype

The third pillar of the CiPA initiative proposes to employ in vitro hiPSC-CMs to confirm the effects of a novel drug predicted by a comprehensive in silico model. hiPSC-CMs and adult ventricular CMs show qualitatively consistent responses to some, but not all drugs (9,20,30). Recently, two computational studies (8,31) quantitatively reported on the main electrophysiological differences between hiPSC-CM and adult ventricular CMs by comparing the Paci 2013 and O'Hara-Rudy models. Paci et al. (22) investigated the discrepancies between hiPSC-CM and adult ventricular CMs by simulating the effects of pharmaceutical block of several membrane currents. They observed that the most relevant differences emerged during the block of $I_{Ca,L}$ and I_{K1} because of the overexpression of I_{NaCa} and a reduced repolarization reserve in hiPSC-CM. Gong and Sobie (31) systematically investigated the differences between hiPSC-CM and adult ventricular CM models and designed a mathematical approach to predict the effect of a drug on adult CMs based on recordings from hiPSC-CMs exposed to the drug. This approach is highly accurate when using in silico models; however, its in vitro validation is not easy to achieve: the availability of adult ventricular cell is scarce, and the variability between hiPSC-CM is likely to decrease the accuracy.

hiPSC-CMs with a mature electrophysiological phenotype are not available yet. Approaches such as increasing I_{K1} via dynamic clamping or ectopic overexpression of the KCNJ2 bring the AP waveform closer to that of an adult human cardiomyocyte. Combining this technique with the mathematical methods to extrapolate findings to adult human cardiomyocytes (such as proposed by Gong and Sobie (31)) may bring us closer to a predictive human cardiomyocyte model.

CONCLUSIONS

In this exploratory in silico study, we addressed the issue of the immature electrophysiological profile of hiPSC-CMs. From the simulations, it can be concluded that 1) the electronic expression of I_{K1} according to Ishihara et al. (19) is able to successfully stop the automaticity of hiPSC-CMs and shows time-dependent properties that may be important for the evaluation of drug safety, and 2) the definition of $G_{K1,\ critical}$ allows researchers to tailor the amount of I_{K1} for each cell, reaching an RMP comparable to adult CMs. $G_{K1,\ critical}$ will be a sensitive parameter during in vitro DC experiments; it will be a sort of a fingerprint that characterizes every cell under investigation. The automatic identification of $G_{K1,\ critical}$ will further facilitate implementation of DC on multichannel automated patch-clamp platforms, overcoming the low throughput that characterizes the combined use of manual patch clamping and DC.

Limitations

The in silico results we reported in our work are based on the hiPSC-CM model published by Paci et al. in 2013 (22). More recently, updated or adapted versions of this model were published (32,33). Adoption of these two new hiPSC-CM computational models is likely to lead to slightly

different values for $G_{K1,\ critical}$ when comparing the six I_{K1} formulations. In this study, we addressed potential hiPSC-CM model dependency of our conclusions by building cell-specific models based on the Paci 2013 model and the approach by Lei et al. (24). Indeed, the identified $G_{K1,\ critical}$ values were slightly different but still very comparable to the values found using the initial Paci 2013 model. Kernik et al. (34) recently published their hiPSC-CM model based on several experimental data sets, collected in different laboratories. Because the model development is different with respect to the aforementioned models, the identification of $G_{K1,critical}$ in that model would bring further information about model dependency and cellular variability. Furthermore, this study used pacing at 1 Hz, a decision that was informed by our earlier work using human stem-cell-derived cardiomyocytes (35,36). Using lower or higher pacing frequencies may affect the observed $G_{K1,\ critical}$ values.

SUPPORTING MATERIAL

Supporting Material can be found online at https://doi.org/10.1016/j.bpj.2019.08.040.

AUTHOR CONTRIBUTIONS

A.F., T.A.B.v.V., M.A.V., and T.P.d.B designed the research. A.F., B.G., and T.P.d.B. performed the research. A.F., B.G., and T.P.d.B. analyzed the data. A.F., B.G., M.A.V., T.A.B.v.V., and T.P.d.B. wrote the manuscript.

ACKNOWLEDGMENTS

The authors thank Dr. Jonathan Cooper (University College London) and Dr. Gary Mirams (the University of Nottingham) for their help with setting up simulations in the Cardiac Electrophysiology Web Lab platform and Dr. Alain van Mil, Iris van Adrichem, and Inge Dokter (University Medical Center Utrecht) for the supply of the iPSC-CMs.

This research is financially supported by MKMD grants with project numbers 114021501 and 114022502 (Netherlands Organization for Health Research and Development).

REFERENCES

1. Kola, I., and J. Landis. 2004. Can the pharmaceutical industry reduce attrition rates? *Nat. Rev. Drug Discov.* 3:711–715.
2. Ferri, N., P. Siegl, …, R. Benghozi. 2013. Drug attrition during pre-clinical and clinical development: understanding and managing drug-induced cardiotoxicity. *Pharmacol. Ther.* 138:470–484.
3. Laverty, H., C. Benson, …, J. Valentin. 2011. How can we improve our understanding of cardiovascular safety liabilities to develop safer medicines? *Br. J. Pharmacol.* 163:675–693.
4. Colatsky, T., B. Fermini, …, N. Stockbridge. 2016. The comprehensive in vitro proarrhythmia assay (CiPA) initiative - update on progress. *J. Pharmacol. Toxicol. Methods.* 81:15–20.
5. Gintant, G., P. T. Sager, and N. Stockbridge. 2016. Evolution of strategies to improve preclinical cardiac safety testing. *Nat. Rev. Drug Discov.* 15:457–471.
6. O'Hara, T., L. Virág, …, Y. Rudy. 2011. Simulation of the undiseased human cardiac ventricular action potential: model formulation and experimental validation. *PLoS Comput. Biol.* 7:e1002061.
7. Paci, M., E. Passini, …, B. Rodriguez. 2017. Phenotypic variability in LQT3 human induced pluripotent stem cell-derived cardiomyocytes and their response to antiarrhythmic pharmacologic therapy: an in silico approach. *Heart Rhythm.* 14:1704–1712.
8. Paci, M., J. Hyttinen, …, S. Severi. 2015. Human induced pluripotent stem cell-derived versus adult cardiomyocytes: an in silico electrophysiological study on effects of ionic current block. *Br. J. Pharmacol.* 172:5147–5160.
9. Goversen, B., M. A. G. van der Heyden, …, T. P. de Boer. 2018. The immature electrophysiological phenotype of iPSC-CMs still hampers in vitro drug screening: special focus on I_{K1}. *Pharmacol. Ther.* 183:127–136.
10. Meijer van Putten, R. M., I. Mengarelli, …, R. Wilders. 2015. Ion channelopathies in human induced pluripotent stem cell derived cardiomyocytes: a dynamic clamp study with virtual IK1. *Front. Physiol.* 6:7.
11. Verkerk, A. O., C. C. Veerman, …, R. Wilders. 2017. Patch-clamp recording from human induced pluripotent stem cell-derived cardiomyocytes: improving action potential characteristics through dynamic clamp. *Int. J. Mol. Sci.* 18:1873.
12. Bett, G. C., A. D. Kaplan, …, R. L. Rasmusson. 2013. Electronic "expression" of the inward rectifier in cardiocytes derived from human-induced pluripotent stem cells. *Heart Rhythm.* 10:1903–1910.
13. Vaidyanathan, R., Y. S. Markandeya, …, L. L. Eckhardt. 2016. IK1-enhanced human-induced pluripotent stem cell-derived cardiomyocytes: an improved cardiomyocyte model to investigate inherited arrhythmia syndromes. *Am. J. Physiol. Heart Circ. Physiol.* 310:H1611–H1621.
14. Wilders, R. 2006. Dynamic clamp: a powerful tool in cardiac electrophysiology. *J. Physiol.* 576:349–359.
15. Ortega, F. A., E. Grandi, …, D. J. Christini. 2018. Applications of dynamic clamp to cardiac arrhythmia research: role in drug target discovery and safety pharmacology testing. *Front. Physiol.* 8:1099.
16. Goversen, B., N. Becker, …, T. P. de Boer. 2018. A hybrid model for safety pharmacology on an automated patch clamp platform: using dynamic clamp to join iPSC-derived cardiomyocytes and simulations of I_{k1} ion channels in real-time. *Front. Physiol.* 8:1094.
17. Lu, G., A. Horvath, …, T. P. de Boer. 2019. Introducing simulated IK1 into human iPSC-cardiomyocytes using dynamic clamp on an automated patch clamp system. *Biophys. J.* 116:99a.
18. Dhamoon, A. S., S. V. Pandit, …, J. M. Anumonwo. 2004. Unique Kir2.x properties determine regional and species differences in the cardiac inward rectifier K+ current. *Circ. Res.* 94:1332–1339.
19. Ishihara, K., N. Sarai, …, S. Matsuoka. 2009. Role of Mg(2+) block of the inward rectifier K(+) current in cardiac repolarization reserve: a quantitative simulation. *J. Mol. Cell. Cardiol.* 47:76–84.
20. Ma, J., L. Guo, …, C. T. January. 2011. High purity human-induced pluripotent stem cell-derived cardiomyocytes: electrophysiological properties of action potentials and ionic currents. *Am. J. Physiol. Heart Circ. Physiol.* 301:H2006–H2017.
21. ten Tusscher, K. H., D. Noble, …, A. V. Panfilov. 2004. A model for human ventricular tissue. *Am. J. Physiol. Heart Circ. Physiol.* 286:H1573–H1589.
22. Paci, M., J. Hyttinen, …, S. Severi. 2013. Computational models of ventricular- and atrial-like human induced pluripotent stem cell derived cardiomyocytes. *Ann. Biomed. Eng.* 41:2334–2348.
23. Ishihara, K., D. H. Yan, …, T. Ehara. 2002. Inward rectifier K(+) current under physiological cytoplasmic conditions in Guinea-pig cardiac ventricular cells. *J. Physiol.* 540:831–841.
24. Lei, C. L., K. Wang, …, L. Polonchuk. 2017. Tailoring mathematical models to stem-cell derived cardiomyocyte lines can improve predictions of drug-induced changes to their electrophysiology. *Front. Physiol.* 8:986.
25. Garny, A., and P. J. Hunter. 2015. OpenCOR: a modular and interoperable approach to computational biology. *Front. Physiol.* 6:26.

26. Cooper, J., M. Scharm, and G. R. Mirams. 2016. The cardiac electrophysiology web lab. *Biophys. J.* 110:292–300.

27. Meijer van Putten, R. M., I. Mengarelli, …, R. Wilders. 2015. Ion channelopathies in human induced pluripotent stem cell derived cardiomyocytes: a dynamic clamp study with virtual IK1. *Front. Physiol.* 6:7.

28. Rocchetti, M., L. Sala, …, A. Zaza. 2017. Elucidating arrhythmogenic mechanisms of long-QT syndrome CALM1-F142L mutation in patient-specific induced pluripotent stem cell-derived cardiomyocytes. *Cardiovasc. Res.* 113:531–541.

29. Britton, O. J., N. Abi-Gerges, …, B. Rodriguez. 2017. Quantitative comparison of effects of dofetilide, sotalol, quinidine, and verapamil between human *ex vivo* trabeculae and *in silico* ventricular models incorporating inter-individual action potential variability. *Front. Physiol.* 8:597.

30. Matsa, E., D. Rajamohan, …, C. Denning. 2011. Drug evaluation in cardiomyocytes derived from human induced pluripotent stem cells carrying a long QT syndrome type 2 mutation. *Eur. Heart J.* 32:952–962.

31. Gong, J. Q. X., and E. A. Sobie. 2018. Population-based mechanistic modeling allows for quantitative predictions of drug responses across cell types. *NPJ Syst. Biol. Appl.* 4:11.

32. Paci, M., R. P. Pölönen, …, J. Hyttinen. 2018. Automatic optimization of an *in silico* model of human iPSC derived cardiomyocytes recapitulating calcium handling abnormalities. *Front. Physiol.* 9:709.

33. Koivumäki, J. T., N. Naumenko, …, P. Tavi. 2018. Structural immaturity of human iPSC-derived cardiomyocytes: in silico investigation of effects on function and disease modeling. *Front. Physiol.* 9:80.

34. Kernik, D. C., S. Morotti, …, C. E. Clancy. 2019. A computational model of induced pluripotent stem-cell derived cardiomyocytes incorporating experimental variability from multiple data sources. *J. Physiol.* 597:4533–4564.

35. Jonsson, M. K., G. Duker, …, T. A. van Veen. 2010. Quantified proarrhythmic potential of selected human embryonic stem cell-derived cardiomyocytes. *Stem Cell Res.* 4:189–200.

36. Jonsson, M. K., M. A. Vos, …, T. A. van Veen. 2012. Application of human stem cell-derived cardiomyocytes in safety pharmacology requires caution beyond hERG. *J. Mol. Cell. Cardiol.* 52:998–1008.

37. Koumi, S., R. Sato, and H. Hayakawa. 1994. Modulation of the delayed rectifier K+ current by apamin in Guinea-pig heart. *Eur. J. Pharmacol.* 261:213–216.

38. Yan, D.-H., and K. Ishihara. 2005. Two Kir2.1 channel populations with different sensitivities to Mg(2+) and polyamine block: a model for the cardiac strong inward rectifier K(+) channel. *J. Physiol.* 563:725–744.

Biophysical Journal, Volume *117*

Supplemental Information

Required G_{K1} to Suppress Automaticity of iPSC-CMs Depends Strongly on I_{K1} Model Structure

Alan Fabbri, Birgit Goversen, Marc A. Vos, Toon A.B. van Veen, and Teun P. de Boer

$G_{K1,critical}$ required for suppression of automaticity of iPSC-CM in dynamic clamp experiments strongly depends on I_{K1} model formulation used

A. Fabbri, B. Goversen, M.A. Vos, T.A.B. van Veen, T.P. de Boer

Supplementary Material

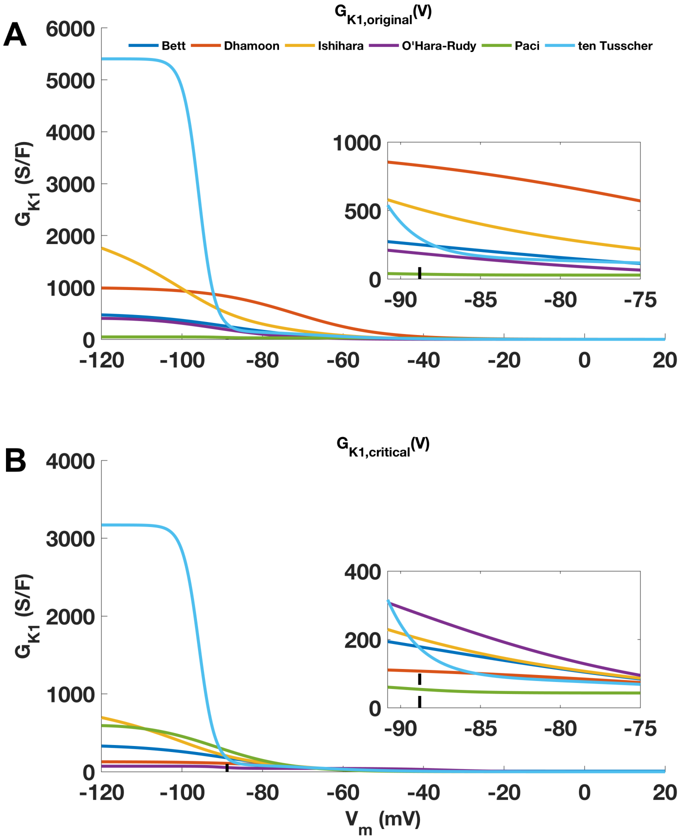

Figure S1: $G_{K1}(V)$ in original (published) and critical conditions
A,B) G_{K1} depends on the membrane potential V_m. The insets magnify the G_{K1} in a voltage range close to the RMP. Larger G_{K1} is in this voltage range causes a hyperpolarized RMP, compare the insets and the table below.

$G_{K1,original}$		$G_{K1,critical}$	
**Model**	**RMP (mV)**	**Model**	**RMP (mV)**
Dhamoon	-88.1	O'Hara-Rudy	-86.8
Ishihara	-87.8	Ishihara	-85.9
Bett	-86.8	Bett	-85.9
ten Tusscher	-86.1	ten Tusscher	-83.7
O'Hara-Rudy	-	Dhamoon	-82.6
Paci	-77.2	Paci	-80.4

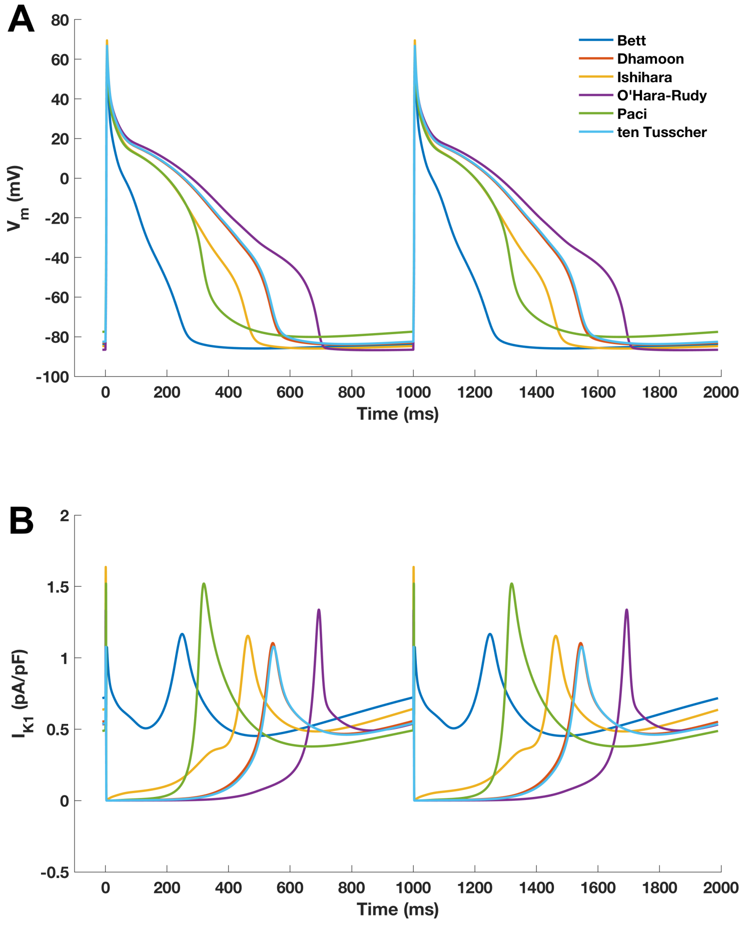

Figure S2: Comparison of I_{K1}formulations for G_{K1}=$G_{K1,critical}$

A) Action potential waveform and B) I_{K1} timecourse in the Paci hiPSC-CM models paced at 1Hz and with G_{K1}=$G_{K1,critical}$. Of note, (i) the models including the I_{K1}formulations according to Dhamoon and ten Tusscher (red and cyan traces) show nearly identical membrane potentials and I_{K1}. (ii) The low I_{K1}density during the diastolic interval is responsible for a less negative membrane potential in Paci

			Bett		Dhamoon		Ishihara		O'Hara-Rudy		Paci		ten Tusscher	
Cell ID	Scaling k NaCa	Scaling gKs	Critical GK1 (S/F)	IK1 avg (pA/pF)	Critical GK1 (S/F)	IK1 avg (pA/pF)	Critical GK1 (S/F)	IK1 avg (pA/pF)	Critical GK1 (S/F)	IK1 avg (pA/pF)	Critical GK1 (S/F)	IK1 avg (pA/pF)	Critical GK1 (S/F)	IK1 avg (pA/pF)
**1**	3.32	139	599.7	0.746	104.2	0.617	828.7	0.698	249.1	0.720	27.7	0.426	2500	0.590
**2**	2.08	129	597.4	0.717	104.2	0.593	818.1	0.670	249.2	0.695	27.9	0.414	2506	0.569
**3**	7.55	125	600.7	0.801	103.6	0.638	831.8	0.735	251.3	0.753	28.0	0.435	2465	0.602
**4**	21.1	467	597.5	0.916	101.1	0.742	824.9	0.882	253.9	0.988	0.0	0.000	2411	0.692
**5**	6.89	270	598.6	0.833	103.5	0.690	827.9	0.792	251.3	0.840	25.7	0.432	2470	0.651
**6**	9.3	211	598.9	0.843	103.6	0.686	830.3	0.793	252.6	0.838	26.3	0.435	2447	0.642
**7**	0.951	37.6	586.7	0.677	104.0	0.557	826.9	0.634	251.8	0.650	29.8	0.399	2513	0.535
**8**	1.54	19.5	590.7	0.684	104.5	0.528	828.6	0.610	252.8	0.597	29.2	0.391	2522	0.507
**9**	1.7	52	591.3	0.694	104.5	0.558	816.4	0.633	251.1	0.644	28.7	0.401	2516	0.535
**10**	1.67	0	590.9	0.680	104.6	0.480	835.6	0.578	252.9	0.511	29.3	0.379	2527	0.459
**11**	0.38	31.5	595.4	0.692	104.6	0.594	824.2	0.666	249.1	0.693	32.0	0.435	2539	0.573
**12**	0.696	18.6	590.2	0.676	104.1	0.558	829.7	0.635	251.0	0.645	30.4	0.401	2520	0.536
**13**	3.05	153	599.2	0.743	104.0	0.616	828.0	0.698	248.7	0.722	27.6	0.425	2498	0.590
**14**	1.28	125	589.0	0.693	104.0	0.582	808.6	0.654	249.9	0.688	28.7	0.407	2502	0.557
**15**	1.19	38.3	585.5	0.680	104.1	0.551	824.9	0.628	252.2	0.639	29.1	0.395	2515	0.529
**16**	3.02	47.3	600.0	0.718	104.9	0.563	823.0	0.641	249.8	0.637	28.2	0.405	2524	0.539
**17**	2.35	27	599.3	0.702	105.0	0.537	820.5	0.616	251.5	0.603	28.8	0.398	2528	0.514
**18**	4.03	138	600.4	0.760	104.3	0.626	828.8	0.709	249.7	0.730	27.5	0.427	2499	0.597
**19**	5.74	28.9	599.8	0.725	105.3	0.517	826.5	0.597	249.8	0.553	28.2	0.389	2545	0.494
**20**	2.35	9.18	598.5	0.695	105.0	0.502	827.5	0.591	252.8	0.547	29.0	0.387	2535	0.480
**21**	3.77	93.7	600.4	0.744	104.5	0.602	828.6	0.683	248.4	0.694	28.1	0.422	2507	0.575
**22**	13.9	288	599.8	0.883	102.3	0.714	819.9	0.833	251.7	0.904	0.0	0.000	2462	0.673
**Mean**			**595.9**	**0.741**	**104.1**	**0.593**	**825.4**	**0.681**	**250.9**	**0.695**	**25.9**	**0.373**	**2502**	**0.565**
**25th percentile**			591.0	0.692	104.0	0.553	823.3	0.629	249.7	0.638	27.6	0.392	2498	0.531
**median**			**598.6**	**0.717**	**104.2**	**0.587**	**827.2**	**0.660**	**251.2**	**0.690**	**28.2**	**0.403**	**2510**	**0.563**
**75th percentile**			599.7	0.756	104.6	0.623	828.7	0.706	252.1	0.728	29.1	0.425	2524	0.595
**min**			585.5	0.676	101.1	0.480	808.6	0.578	248.4	0.511	0.0	0.000	2411	0.459
**max**			600.7	0.916	105.3	0.742	835.6	0.882	253.9	0.988	32.0	0.435	2545	0.692

Table S1: Cell specific models: $G_{K1,critical}$ and average I_{K1} during late depolarization

We identified $G_{K1,critical}$ for each cell specific model (n=22) including the six I_{K1} formulations. The results of the bisection search are collected in the $G_{K1,critical}$ columns. I_{K1} avg represents the average I_{K1} flowing during the last 50 ms before the next stimulus when the cells are paced at 1 Hz.

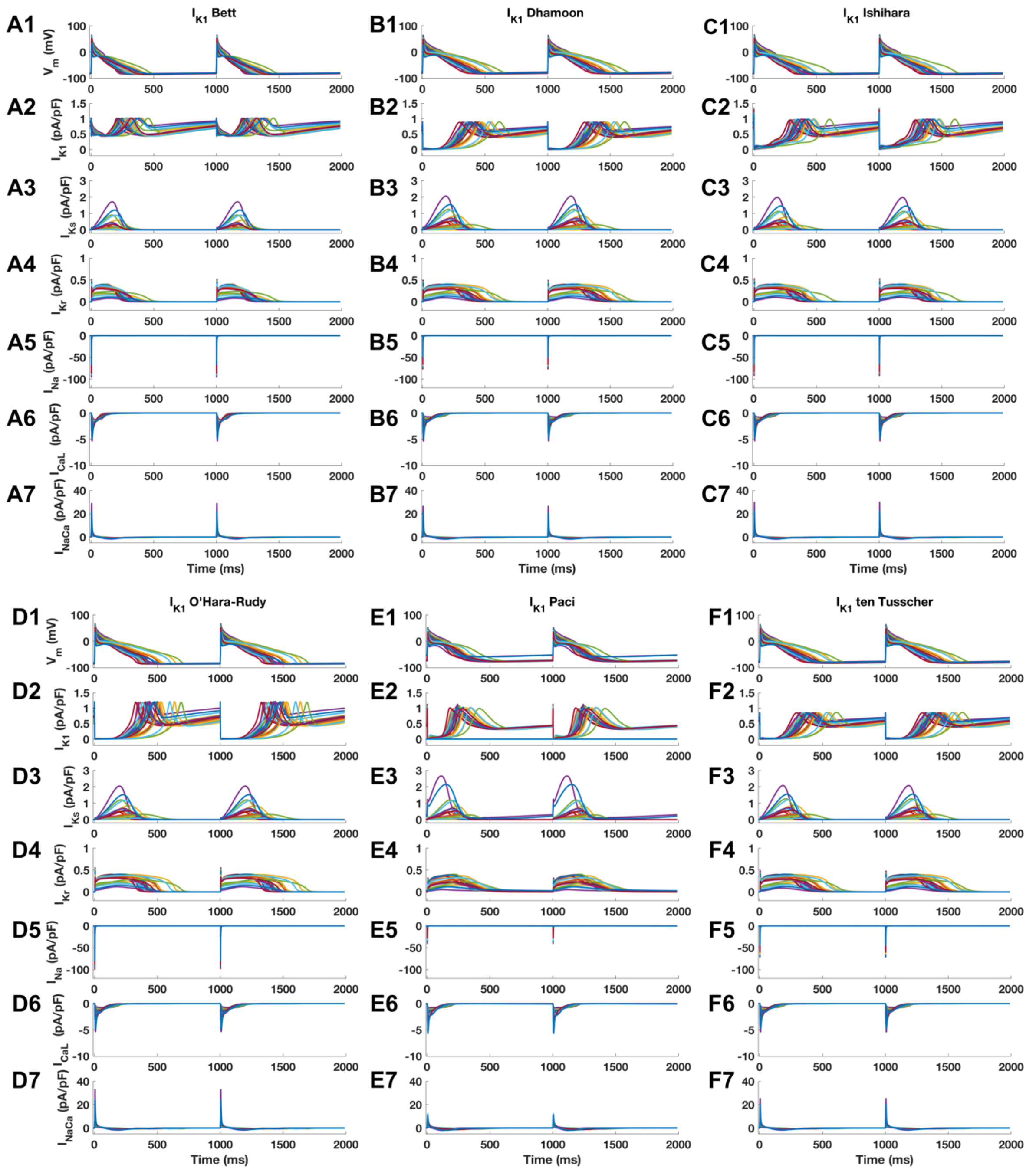

Figure S3: Membrane potential and main currents in the cell specific models

Membrane potential, I_{K1}, I_{Ks}, I_{Kr}, I_{Na} ,I_{CaL} and I_{NaCa} of the 22 cell specific models for each I_{K1} formulation. G_{K1} was set to the critical value identified running the bisection algorithm for each cell model

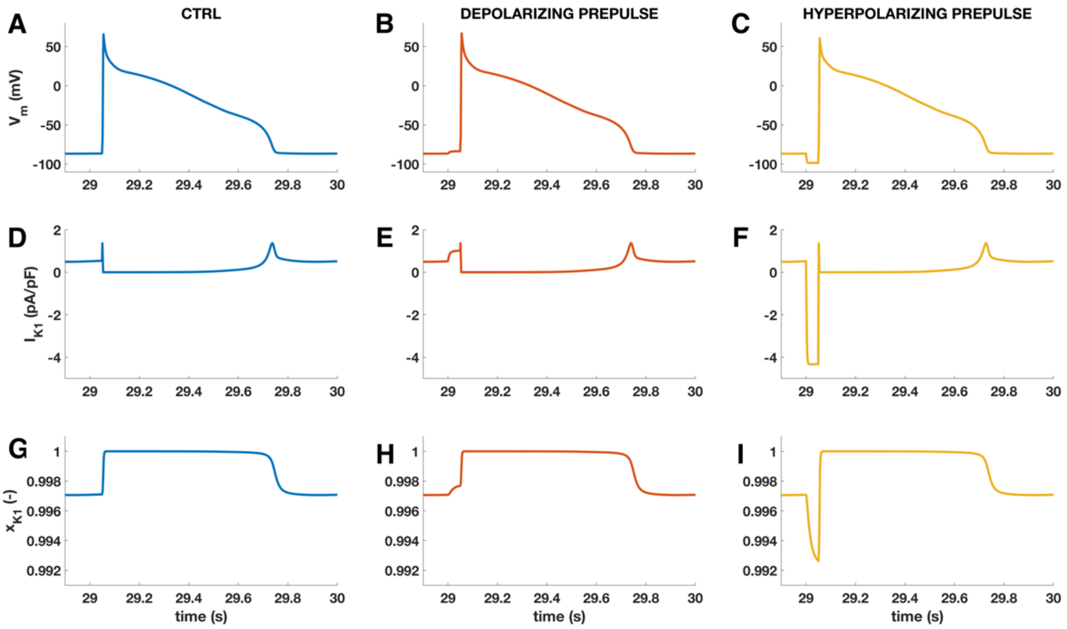

Figure S4: time dependency of I_{K1} in the O'Hara-Rudy formulation.
Membrane potential (A-C), I_{K1} density (D-F), and inactivation variable x_{K1} (G-I) time courses in CTRL conditions (first column) and in response to depolarizing and hyperpolarizing prepulses (second and third colums, respectively).
Panels G-I show the first order kinetics of x_{K1}, but since it's varying between 1 and 0.993, it can be considered negligible, even in CTRL conditions.

	Last APD_{90}				
**Model**	**CTRL**	**DEP**	**$\Delta APD_{90,Dep}$ (%)**	**HYPER**	**$\Delta APD_{90,Hyper}$ (%)**
**Bett**	237	240	1.3	232	-2.1
**Dhamoon**	540	544	0.7	522	-3.5
**Ishihara**	459	489	6.5	384	-16.3
**O'Hara-Rudy**	678	681	0.4	668	-1.5
**Paci**	356	357	0.3	339	-4.8
**ten Tusscher**	545	549	0.7	538	-1.3

Table S2: Preconditioning prepulse protocol
APD_{90} in the last AP of a cycle of thirty 1 Hz paced APs in CTRL conditions, in response to a depolarizing (DEP) or hyperpolarizing (HYPER) prepulse.

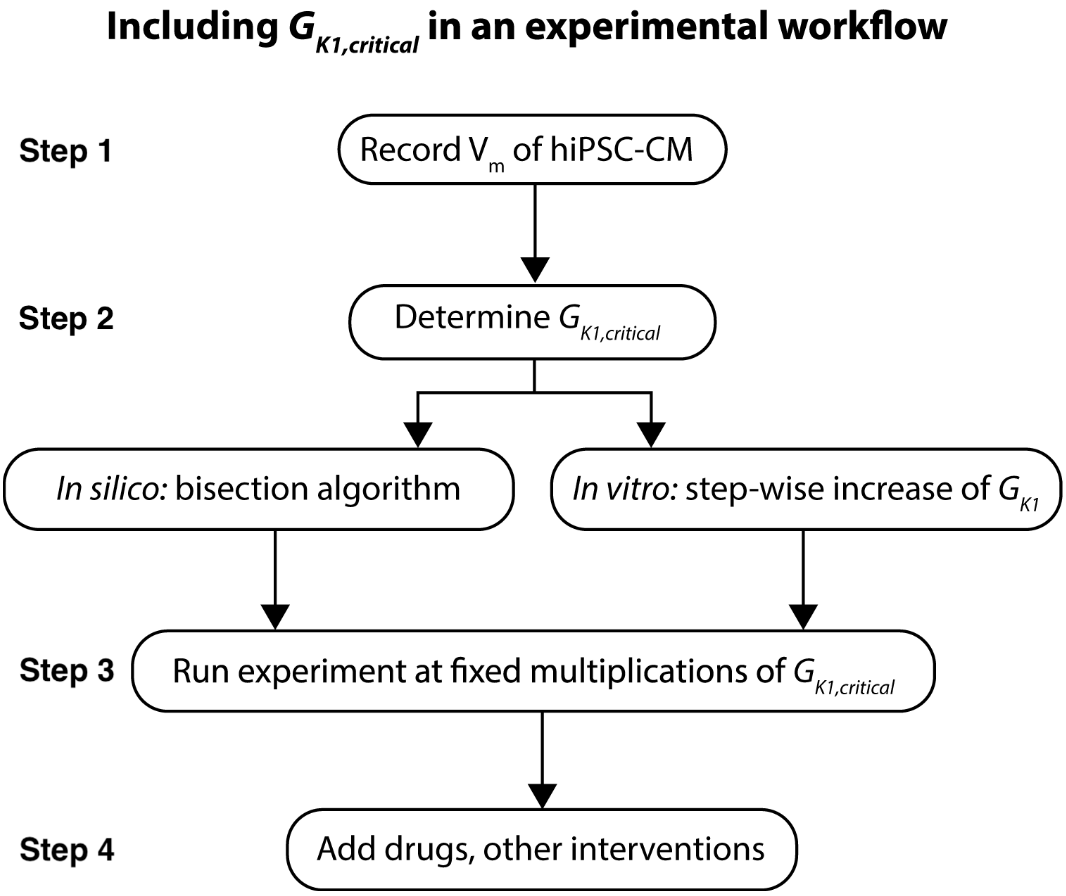

Figure S5: Including $G_{K1,critical}$ in an experimental workflow
Proposed steps in dynamic clamp or in silico experiments that include determination of $G_{K1,critical}$.
Step 1. Membrane potential is measured from round shaped and spontaneously beating hiPSC-CMs.
Step 2. *In silico* simulations allow to identify precisely $G_{K1,critical}$ for a cell specific or an average cell model. In an *in* vitro experiment, the bisection algorithm would take too long, and $G_{K1,critical}$ can instead be estimated with a protocol testing at step-wise increasing or decreasing G_{K1} values.
Step 3. Running subsequent experiments at multiplication of $G_{K1,critical}$ helps to reduce variation between cells.
Step 4. Measuring the AP while a drug is administered gives information about the efficacy or safety of the compound under investigation. Combination with results from a computational model could provide a mechanistic interpretation of what is observed during *in vitro* experiments

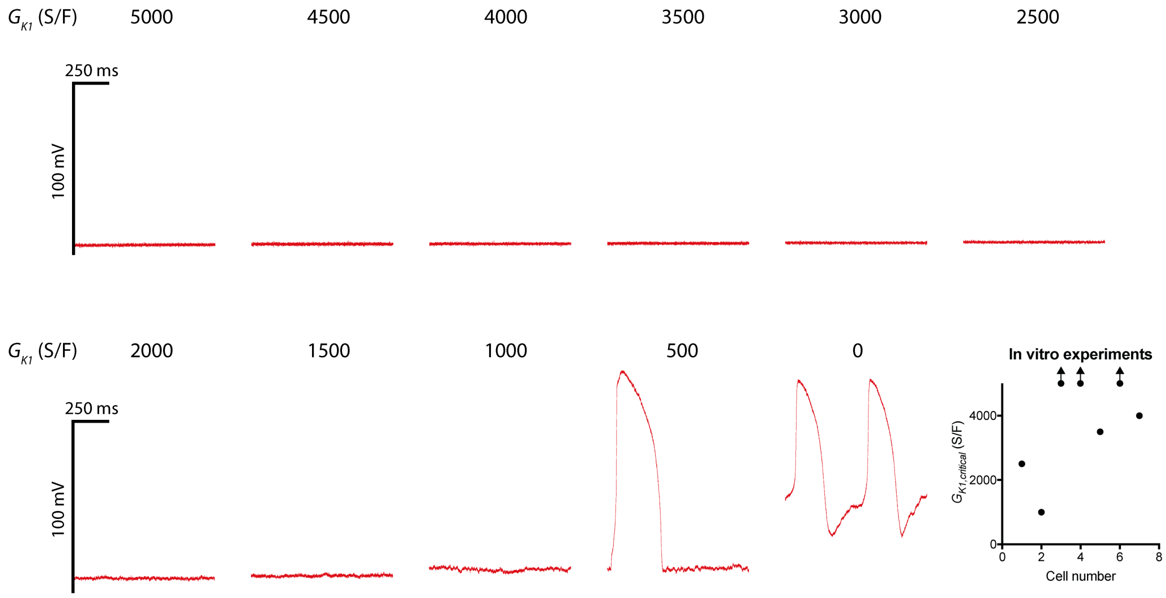

Figure S6: Determining $G_{K1,critical}$ in a dynamic clamp experiment

Main figure: In a set of dynamic clamp experiments using the Ishihara formulation, I_{K1} was injected using G_{K1} values starting at 5000 S/F, which was decreased with 500 S/F steps to 0 S/F. $G_{K1,critical}$ was defined as the minimal G_{K1} required for maintaining quiescence, in this example (Cell 2 in inset) $G_{K1,critical}$ was 1000 S/F. *Inset:* Summary plot of $G_{K1,critical}$ obtained in 7 cells. In the cells indicated with the arrows, the maximum G_{K1} tested did not completely suppress all activity, i.e. $G_{K1,critical}$ was slightly higher. The average $G_{K1,critical}$ in the 4 cells in which we obtained quiescence was 2750 ± 660 S/F.
